# Development of a Robust Diffuse Reflectance Infrared Fourier Transform Spectroscopy (DRIFTS) Cell for Elucidating Reaction Mechanisms in Nonthermal Plasma Catalysis

**DOI:** 10.1002/smtd.202501403

**Published:** 2025-11-20

**Authors:** Jiangqi Niu, Shaowei Chen, Yi Chen, Jinyan Zhang, Guanting Zhou, Huiji Yu, Qingyang Lin, Tianqi Liu, Shanshan Xu, Zifu Li, Jianguo Huang, Huanhao Chen, Xiaolei Fan

**Affiliations:** ^1^ Wenzhou Key Laboratory of Novel Optoelectronic and Nano Materials Institute of Wenzhou Zhejiang University Wenzhou 325006 China; ^2^ Department of Chemical and Biomolecular Engineering National University of Singapore Engineering Drive 4 Singapore 117585 Singapore; ^3^ State Key Laboratory of Materials‐Oriented Chemical Engineering College of Chemical Engineering Nanjing Tech University Nanjing 211816 China; ^4^ Institute of Thermal Science and Power Systems College of Energy Engineering Zhejiang University Hangzhou 310027 China; ^5^ Department of Chemical Engineering School of Engineering The University of Manchester Oxford Road Manchester M13 9PL UK; ^6^ Zhejiang Tihe Instrument Co., Ltd. 701 Huzhou Street Hangzhou 310015 China; ^7^ Ningbo China Beacons of Excellence Research and Innovation Institute University of Nottingham Ningbo China 211 Xingguang Road Ningbo 315048 China

**Keywords:** diffuse reflectance infrared Fourier transform spectroscopy (DRIFTS), in situ diagnostics, mechanisms, nonthermal plasma catalysis

## Abstract

Applying diffuse reflectance infrared Fourier transform spectroscopy (DRIFTS) to nonthermal plasma catalysis is important to gain mechanistic information to advance the electrified technology. However, conventional DRIFTS cells are often suboptimal, exhibiting unstable, non‐uniform discharges and failing to replicate the plasma characteristics of practical reactors such as dielectric barrier discharge (DBD) systems. This study devises a dome‐type DRIFTS flow cell that enables stable glow discharge, closely emulating the electric field distribution and flow dynamics of DBD reactors. The dome cell exhibits excellent operational stability over extended durations (>1 h) and under various plasma conditions (e.g., different excitation modes and gas switching), while delivering high‐fidelity IR signals. Using the dome cell, *operando* DRIFTS studies of plasma catalytic CO_2_ methanation under pulse excitation are conducted. The results reveal that, for a Ni/MgAlO_x_ catalyst, i) the surface reaction mainly follows the Langmuir–Hinshelwood mechanism with formate hydrogenation as the rate‐determining step, and ii) the Eley‐Rideal/Langmuir–Rideal mechanism indeed exists under plasma conditions but contributes marginally. This robust DRIFTS platform provides a reliable in situ and/or *operando* diagnostic tool for plasma catalytic systems, while offering mechanistic insights essential for rational catalyst/system design and optimization.

## Graphical abstract

A dome‐type *operando* DRIFTS cell is developed to enable stable, uniform glow discharges that mimic dielectric barrier discharge (DBD) reactors. In plasma‐catalytic CO_2_ methanation, this design allows real‐time mechanistic tracking of surface reactions under gas switching conditions, revealing reaction pathways. The platform provides high‐fidelity insights essential for advancing plasma catalysis.

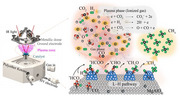

## 1 Introduction

Nonthermal plasma (NTP) assisted catalytic reactions (or plasma catalysis)^[^ ^1^, ^2^ ^]^ is a promising technology for exploiting renewable energy to drive sustainable chemical conversions such as carbon dioxide (CO_2_) hydrogenation^[^ ^3^ ^]^ and nitrogen fixation.^[^ ^4^ ^]^ In plasma catalysis, non‐equilibrium electron collisions and ion–molecule interactions^[^ ^5^ ^]^ generate electronically and vibrationally excited species^[^ ^6^ ^]^ (Figure S1, Supporting Information) to participate in surface interactions. Owing to this unique activation process in gas discharge, both the Langmuir–Hinshelwood (L–H) and Eley–Rideal/Langmuir–Rideal (E–R/L–R) mechanisms may operate simultaneously under plasma conditions,^[^ ^7^, ^8^, ^9^, ^10^ ^]^ leading to reaction pathways distinct from those in conventional thermal catalysis. To advance such electrified hybrid technologies, a mechanistic understanding of complex phenomena in plasma catalytic systems, especially surface reactions, is indispensable. In situ diffuse reflectance infrared Fourier transform spectroscopy (DRIFTS) is commonly employed to probe surface intermediates and elucidate reaction mechanisms in plasma catalytic systems.^[^ ^11^, ^12^, ^13^, ^14^ ^]^ However, obtaining reliable and high‐quality infrared (IR) spectral data critically depends on maintaining stable plasma operation under varying discharge conditions.

Plasma catalysis is typically studied in dielectric barrier discharge (DBD) reactors,^[^ ^15^ ^]^ which sustain uniform plasma morphology while preventing arc formation^[^ ^16^ ^]^ (a phenomenon that otherwise causes excessive local heating and gas expansion). Therefore, during DRIFTS experiments, the plasma discharge characteristics within the DRIFTS flow cells must replicate discharge characteristics of DBD reactors, including the type and density of plasma‐induced species, electron temperature and density, and discharge stability. Such resemblance is vital for establishing meaningful correlations between reaction mechanisms (gained from DRIFTS) and catalyst performance (observed in practical DBD reactors). Despite progress in in situ DRIFTS applications, current DRIFTS cell designs often exhibit discharge characteristics that diverge significantly from those in DBD reactors. For instance, jet‐ and pin‐type DRIFTS cells have been employed to simulate DBD‐like environments.^[^ ^17^ ^]^ These configurations offer valuable insights, as they mimic some features of single‐ or dual‐stage DBD systems, and in some cases, minimize the perturbation of plasma properties by the catalyst.^[^ ^17^, ^18^ ^]^ Nevertheless, these configurations typically produce strong filamentary or arc‐like discharges, resulting in poor long‐term stability. Consequently, in situ data collected under such unstable plasma conditions may not accurately represent catalyst behavior in practical DBD reactors, potentially leading to misleading mechanistic interpretations.

**Figure 1:**
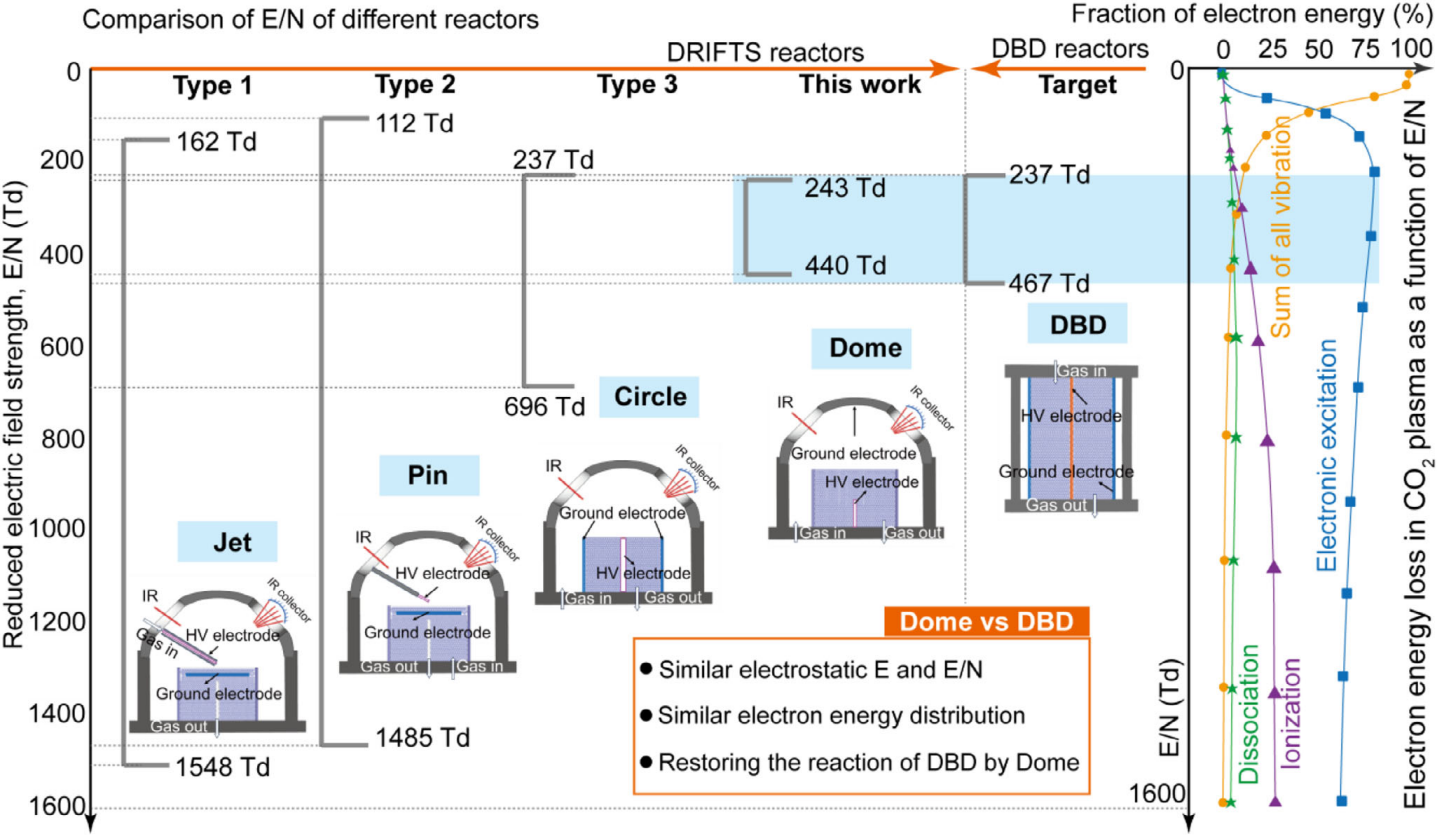
Reduced electric field strength (E/N) for different in situ DRIFTS flow cells and DBD reactors used in plasma catalysis research. The fraction of electron energy allocated to different electron collision processes in CO_2_ plasma, including vibrational and electronic excitation, ionization, and dissociation, as a function of E/N.^[^ ^34^ ^]^ The orange rectangle shows the similarity between the electric field characteristics of the dome‐type cell designed in this work and the DBD reactor.

Current DRIFTS flow cell designs for plasma catalysis (Table S1, Supporting Information) generally adopt jet,^[^ ^13^, ^17^, ^19^, ^20^ ^]^ pin,^[^ ^21^, ^22^, ^23^, ^24^ ^]^ and circle type^[^ ^7^, ^25^, ^26^, ^27^, ^28^, ^29^, ^30^, ^31^ ^]^ electrode configurations, illustrated in **Figures** **1** and S2 (Supporting Information). These geometries lead to distinct distributions of the reduced electric field (E/N, Figure 1, using CO_2_ plasma as the example), a key parameter describing the effectiveness of electron acceleration by electric field between collisions with neutral gas molecules, dictating the energy partitioning among vibrational excitation, ionization, and dissociation processes.^[^ ^32^ ^]^ As shown in Figure 1, DBD reactors feature a relatively narrow E/N distribution (237–467 Td),^[^ ^33^ ^]^ ensuring uniform energy partitioning of excited reactive species across the catalyst bed.^[^ ^34^ ^]^ Conversely, E/N distributions in the conventional DRIFTS cells are much broader, (162–1548 Td for jet‐type, 112–1485 Td for pin‐type, and 237–696 Td for circle‐type), resulting in significant deviations in plasma‐induced species and reactivity compared to DBD systems.^[^ ^35^ ^]^ In addition, excessively high local E/N values in these cells can lead to plasma instability, catalyst displacement, and arc formation, particularly during prolonged operation. These limitations highlight the need for optimized DRIFTS cell designs that more faithfully replicate DBD plasma characteristics.

Here, we report the design and development of a dome‐type DRIFTS cell (FANNE, model: PCT‐DM‐G01), that features a metallic dome as the ground electrode and an embedded high‐voltage (HV) electrode (positioned within the catalyst bed). This configuration suppresses extreme local electric fields and enables stable, uniform plasma generation. The dome‐type cell exhibits discharge characteristics closely resembling those of DBD reactors, ensuring comparable plasma–catalyst interactions. It also demonstrates excellent operational stability over multiple plasma on/off cycles, robust performance under various excitation conditions, and rapid responsiveness to dynamic gas compositions. Importantly, using this dome‐type cell, we conducted in situ DRIFTS studies of plasma‐catalytic CO_2_ methanation and revealed that the surface reaction primarily proceeds via the L–H mechanism, with hydrogenation of surface formates as the rate‐determining step, while the E–R/L–R pathway contributes negligibly. Hence, the newly developed dome‐type DRIFTS flow cell offers a robust and reliable platform for in situ and *operando* mechanistic investigations of plasma–catalytic reactions.

## 2 Results and Discussion

### 2.1 Dome DRIFTS Cell with Electric/Flow Characteristics Representative of DBD Reactors

A mechanistic understanding of catalytic conversions under realistic plasma conditions demands a DRIFTS flow cell capable of sustaining stable discharge and maintaining operational robustness across different discharge intensities. To this end, we systematically compared the electric characteristics of several commercial DRIFTS cells with those of our dome‐type cell and DBD reactors. The dome‐type cell, which employs a metallic dome as the ground electrode and an embedded HV electrode (Figure S2a, Supporting Information), was specifically engineered to reproduce the electric and flow field distributions characteristic of DBD systems. Detailed structural design and fabrication procedure are described in Experimental Section–Cell Design and Manufacturing. Electrostatic field simulations conducted using Ansys Maxwell (Experimental Section–Simulation Methods, Figure S2b, Supporting Information) revealed that embedding the HV electrode (insulated by a sealed‐end quartz tube) within the catalyst bed effectively suppresses tip‐induced field enhancement. This configuration provides abundant microdischarge pathways,^[^ ^36^ ^]^ facilitating spatial dispersion of charged species and preventing rapid development of electron avalanches in the gas phase. The metallic dome electrode enclosing the discharge zone acts as a quasi‐equipotential surface, promoting a uniform electric field distribution (strength of ≈2 × 10^7^ V m^−1^). This uniformity supports homogeneous streamer propagation through both the catalyst bed and the overlying gas‐phase region, enabling stable glow plasma generation and minimizing arc discharges. Conversely, conventional DRIFTS cell designs (of the circle‐/pin‐/jet‐type) employ sharp‐tip electrodes with small curvature radii that are directly exposed to the gas phase. These geometries concentrate the local electric field, initiating intense electron avalanches near the electrode surface and encouraging unrestricted streamer propagation (Figure S2c–h, Supporting Information). Consequently, such configurations often exhibit non‐uniform electric fields, unstable discharge behaviors, transitions between glow and arc modes, excessive Joule heating, and poor operational stability.^[^ ^37^ ^]^

The dome‐type design effectively mitigates these limitations by suppressing local field enhancement, thereby establishing a uniform electric field comparable to that of DBD reactors, as confirmed by simulated electrostatic field maps (Figure S2i, Supporting Information). Comparison of the calculated E/N distributions near the catalyst bed surface (along Line A in Figure S2b,d,f,h, Supporting Information) demonstrates that the dome cell exhibits a similar and uniform E/N profile to that of DBD reactors, in contrast to the distributions found in conventional cells (Figure S2j, Supporting Information). Computational fluid dynamics (CFD) simulations using *Ansys Fluent* (Experimental Section–Simulation Methods) further revealed that the dome‐type DRIFTS cell replicates the flow field characteristics of DBD reactors. Both systems display comparable gas velocities of ≈0.2 m s^−1^ through the catalyst beds at a flowrate of 90 mL min^−1^ (Figure S3a,b, Supporting Information), and comparable low Reynolds numbers at <80 mL min^−1^ (Figure S3c, Supporting Information), and these results indicate that the dome‐type configuration not only reproduces the electrical characteristics but also emulates the hydrodynamic environment of DBD reactors, thereby providing comparable gas‐solid mass transfer conditions^[^ ^38^ ^]^ and reactant–catalyst interaction dynamics.^[^ ^39^ ^]^

### 2.2 Stable Glow Discharge in the Dome DRIFTS Cell

**Figure 2:**
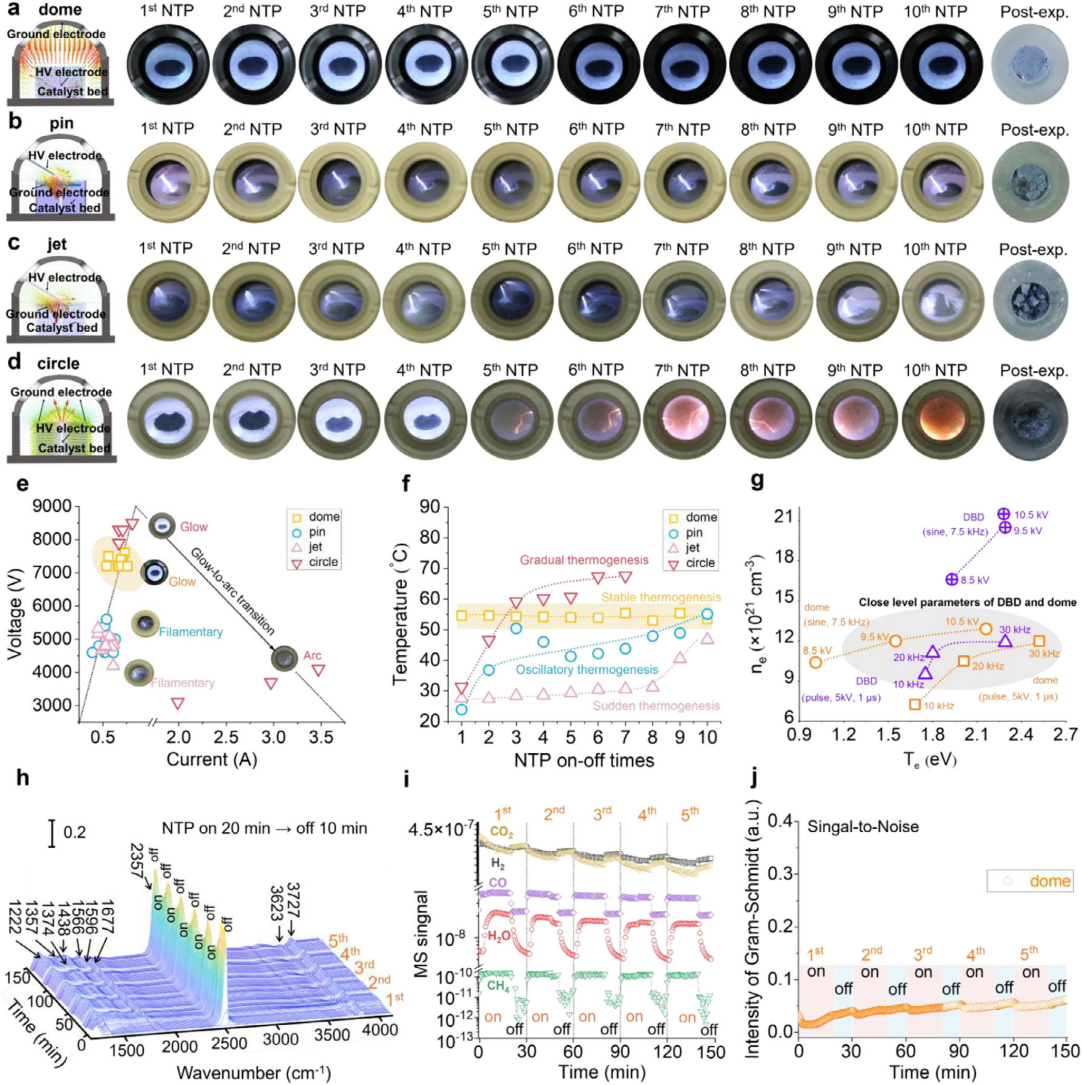
Schematic illustration of electric field vector distribution, plasma morphology (over 10 plasma on/off cycles) for a) dome, b) pin, c) jet, and d) circle DRIFTS flow cells, with the corresponding post‐reaction catalyst bed images. e) Voltage–current characteristics of the four cells. f) Bulk cell temperature evolution during cyclic tests (measured by IR camera and averaged over three readings). g) *n*_e_‐*T*_e_ plots of the dome cell and a DBD under different sine/pulse excitation conditions. h) In situ DRIFTS spectra from the dome cell across 5 plasma on/off cycles. i) Corresponding MS signals at the outlet of the dome cell. j) Gram‐Schmidt intensity tracking of the IR signal during plasma cycling. (Conditions for (h–j): pulse excitation at 5 kV, 20 kHz, 1 µs; DRIFTS feed: 95 vol% Ar + 1 vol% CO_2_ + 4 vol% H_2_ at 1 atm, pCO_2_ = 0.01 atm, pH_2_ = 0.04 atm; DBD feed: 20 vol% Ar + 20 vol% CO_2_ + 60 vol% H_2_ at 1 atm, pCO_2_ = 0.20 atm, pH_2_ = 0.60 atm; total flowrate = 50 mL min^−1^; bulk cell temperature during DRIFTS ≈54 °C; plasma cycles: 20 min on with 10 min off, repeated).

The dome‐type cell exhibits excellent discharge stability and operational robustness, as demonstrated by cyclic plasma on/off testing (using a discharge gas mixture of 95 vol% Ar, 1 vol% CO_2_, and 4 vol% H_2_, coupled with mass spectrometry, MS). Throughout repeated cycles, the cell maintained consistent discharge behavior, characterized by sustained glow discharge, an intact catalyst bed, and highly stable DRIFTS‐MS response signals. Discharge morphologies under both sine and pulse excitations in different inert gas environments (Ar, He, Ar+CO_2_+H_2_, and He+CO_2_+H_2_) are summarized in Figure S4 (Supporting Information) (all measurements at atmospheric pressure). Compared to Ar, plasmas in He exhibit fully diffuse morphologies with minimal filament formation, attributable to helium's lower Townsend ionization coefficient, which suppresses local electron avalanches and favors diffuse discharges. Under sine excitation, the discharges tended to exhibit filamentary features, whereas pulse excitation produced stronger yet spatially uniform discharges. The latter arises because microsecond‐pulse operation preferentially accelerates electrons while minimizing the acceleration of heavy particles, thus reducing gas heating and filamentary instabilities. Owing to these advantages, pulse excitation is widely recognized for its superior energy efficiency compared to continuous modes.^[^ ^40^ ^]^ Accordingly, all cyclic tests in the following studies were conducted under pulse excitation using different DRIFTS cell designs (**Figure** **2**; details in Experimental Section–Methods for Plasma Discharge Diagnostics and Catalytic Performance Evaluation in the Dome Cell). As shown in Figure 2a, the dome cell sustained a homogeneous glow discharge throughout operation, as evidenced by its diffuse discharge morphology, milliampere‐level voltage/current (V/I) characteristics (Figure 2e, which is consistent with the criterion for this discharge regime)^[^ ^41^ ^]^ and stable bulk temperature (at ≈54 °C, measured by IR camera and averaged over three readings, Figure 2f; Figure S5, Supporting Information). Importantly, the glow plasma state is maintained throughout the cyclic tests, confirming excellent discharge stability. After ten cycles (total plasma‐on duration of ≈200 min), the catalyst bed remains macroscopically intact with no signs of powder ejection or bed inhomogeneity, as shown in the post‐experiment image (Figure 2a). The stability ensures consistent optical paths during in situ DRIFTS analysis, preventing spectral artifacts such as peak distortion, baseline drift, or signal attenuation (issues that often arise from instability in plasma cells). Conversely, the pin‐type (Figure 2b,e) and jet‐type (Figure 2c,e) cells exhibited strongly filamentary or arc‐like discharges due to sharp electrode geometries that promote intense local field enhancement. These unstable discharges caused substantial temperature fluctuations within narrow discharge channels during cyclic operation (blue circles and pink up‐pointing triangles in Figure 2f and Figures S6 and S7, Supporting Information), leading to gas turbulence and physical ejection of catalyst particles. Similarly, the circle‐type cell (Figure 2d,e) initially maintained stable operation for up to four cycles but subsequently transitioned to arc discharge due to localized electric field enhancement at the HV electrode surface. This transition led to thermal accumulation, with the bulk temperature rising from 31.3 °C in the first cycle to 60.2 °C by the fourth cycle (Figure 2f; Figure S8, Supporting Information). The resulting temperature increase enlarged the electron mean free path (λ ∝ T^1/2^, where *T* is the bulk temperature) and triggered a glow‐to‐arc transition.^[^ ^42^ ^]^ This instability ultimately caused severe catalyst ejection and discharge failure.

Optical emission spectroscopy (OES) measurements were conducted on both the dome‐type DRIFTS cell and a benchmark DBD reactor under sine and pulse excitation to compare their plasma characteristics (Figures S8 and S9, Supporting Information). The recorded spectra were used to calculate key plasma parameters, i.e., electron temperature (*T*_e_)^[^ ^43^ ^]^ and electron density (*n*_e_),^[^ ^44^ ^]^ as detailed in Note S1 and Table S2, Supporting Information. These parameters reflect the energy state of electrons, which governs the generation of reactive species and thus influences plasma–catalyst interactions. Despite the compositional difference in gas feeds (caption of Figures S9 and S10, Supporting Information), under pulse excitation, both systems exhibit comparable *T*_e_ and *n*_e_ values (Figure 2g): for the dome cell, 2.52–11.68 eV, (0.73–1.20) × 10^22^ cm^−3^; for the DBD reactor, 1.75–2.29 eV and (0.95–1.19) × 10^22^ cm^−3^. In contrast, under sine excitation, significant discrepancies in *T*_e_ and *n*_e_ are observed. The difference arises from the longer acceleration time of heavy particles in sine excitation (66.7 µs half‐cycle) compared to pulse excitation (1 µs pulse width), which enhances ion bombardment at the electrode and catalyst surfaces (the γ effect).^[^ ^45^ ^]^ This process promotes secondary electron emission and collisional ionization, leading to higher *n_e_* values under sine excitation. Overall, these results confirm that under pulse excitation (5 kV, 1 µs, 10–30 kHz), the dome‐type DRIFTS cell reproduces the plasma characteristics of DBD reactors with high fidelity. This capability establishes the necessary foundation for meaningful mechanistic correlations between in situ DRIFTS data and catalytic performance observed in DBD systems.

The dome cell further demonstrated excellent stability during in situ DRIFTS‐MS studies of CO_2_ hydrogenation over a 5 wt.% Ni/MgAlO_x_ catalyst. Catalyst synthesis, characterization, and performance results in DBD systems are presented in Figures S11–S15 (Supporting Information) and discussed in Experimental Section–Methods for Plasma Discharge Diagnostics and Catalytic Performance Evaluation in the Dome Cell and Note S2 (Supporting Information). The in situ DRIFTS spectra (recorded using the method described in Experimental Section–Methods for Plasma Discharge Diagnostics and Catalytic Performance Evaluation in the Dome Cell) show the periodic patterns during plasma on/off cycles (Figure 2h), confirming the reliability of the system. The experiment comprised five cycles, each with 20 min of plasma‐on time followed by 10 min plasma‐off to reach steady‐state conditions. The recurring IR features correspond to surface carbonate (^*^CO_3_^2−^, 1529, 1357 cm^−1^),^[^ ^46^ ^]^ bicarbonate (^*^HCO_3_^−^, 1677, 1222, 1438 cm^−1^),^[^ ^47^ ^]^ formate (^*^HCOO, 1565, 1374 cm^−1^),^[^ ^48^ ^]^ and hydroxyl groups (^*^OH, 3623, 3727 cm^−1^),^[^ ^49^ ^]^ as well as gas‐phase CO (2188 cm^−1^).^[^ ^3^ ^]^ Periodic, reversible variations in the CO_2_ band at 2357 cm^−1^ reflect CO_2_ consumption during plasma‐on periods (Figure 2h; Table S3, Supporting Information).

Complementary MS analysis of the outlet gas (Figure 2i) revealed recurring MS signals corresponding to CH_4_ (m/z = 15), CO (m/z = 28), and H_2_O (m/z = 18), further confirming the occurrence of CO_2_ hydrogenation in the dome cell (Note that these MS data are qualitative and not directly comparable to conversion data in Figure S15, Supporting Information). To quantitatively assess discharge stability and signal integrity, the Gram–Schmidt method was applied to the IR spectral dataset to obtain integrated IR intensities over time.^[^ ^50^ ^]^ The resulting Gram–Schmidt integrated intensity (Figure 2j) shows a slight upward drift with increasing time‐on‐stream, attributable to gradual accumulation of surface ^*^CO_3_^2−^, ^*^HCO_3_^−^, and ^*^HCOO species that marginally enhance the integrated IR signal. Because the method integrates across the full spectral range, this slow buildup manifests as a steady increase. Importantly, no significant fluctuations were observed (standard deviation ≈9.06 × 10^−3^), confirming that i) the presence of plasma on the catalyst surface does not interfere with IR beam path, and ii) abrupt heating and/or gas expansion (typically caused by plasma mode transitions) are absent in the dome cell, and thus high‐quality DRIFTS data are obtained. These findings, consistent with the sustained glow discharge behavior observed earlier (Figure 2a), unequivocally demonstrate that the dome‐type DRIFTS cell enables stable, reproducible in situ diagnostics under realistic plasma conditions, providing a reliable platform for mechanistic investigations of plasma–catalytic CO_2_ hydrogenation.

### 2.3 Stable Operationality of the Dome DRIFTS Cell Under Different Glow Intensities

The dome‐type DRIFTS cell enables robust and stable plasma operation across a broad range of glow intensities, reflecting its ability to balance discharge load and dissipate energy efficiently. This is evidenced by constant instantaneous powers over 60 min of continuous operations (Figure S16a, Supporting Information, CO_2_ hydrogenation over Ni/MgAlO_x_) under different pulse excitation conditions (weak, 12 kHz, 2 µs; moderate, 25 kHz, 3 µs; strong, 30 kHz, 6 µs). The excellent stability, especially under strong excitation conditions, highlights the intrinsic ability of the dome cell to redistribute plasma energy uniformly throughout the cell space. This performance stems from the synergistic effect between the porous catalyst bed and the metallic dome electrode (as discussed above, Figure S2b, Supporting Information), which together facilitate even discharge distribution and efficient energy dissipation, thereby ensuring long‐term operational durability under different plasma intensities. In contrast, conventional DRIFTS cell configurations exhibit markedly lower tolerance to high discharge energies. The pin‐type cell (Figure S16b, Supporting Information) undergoes a rapid glow‐to‐arc transition under moderate excitation, while the jet and circle cells (Figure S16c,d, Supporting Information) experience similar transitions under strong excitation, resulting in operational failure. These instabilities originate from field‐concentrating electrode geometries, where sharp high‐voltage tips exposed to the gas phase (Figure S2d,f,h, Supporting Information) generate localized ionization, collision, and charge accumulation. At elevated excitation energies, discharge propagation preferentially follows these localized high‐field regions, ultimately triggering arc formation and discharge instability.

**Figure 3:**
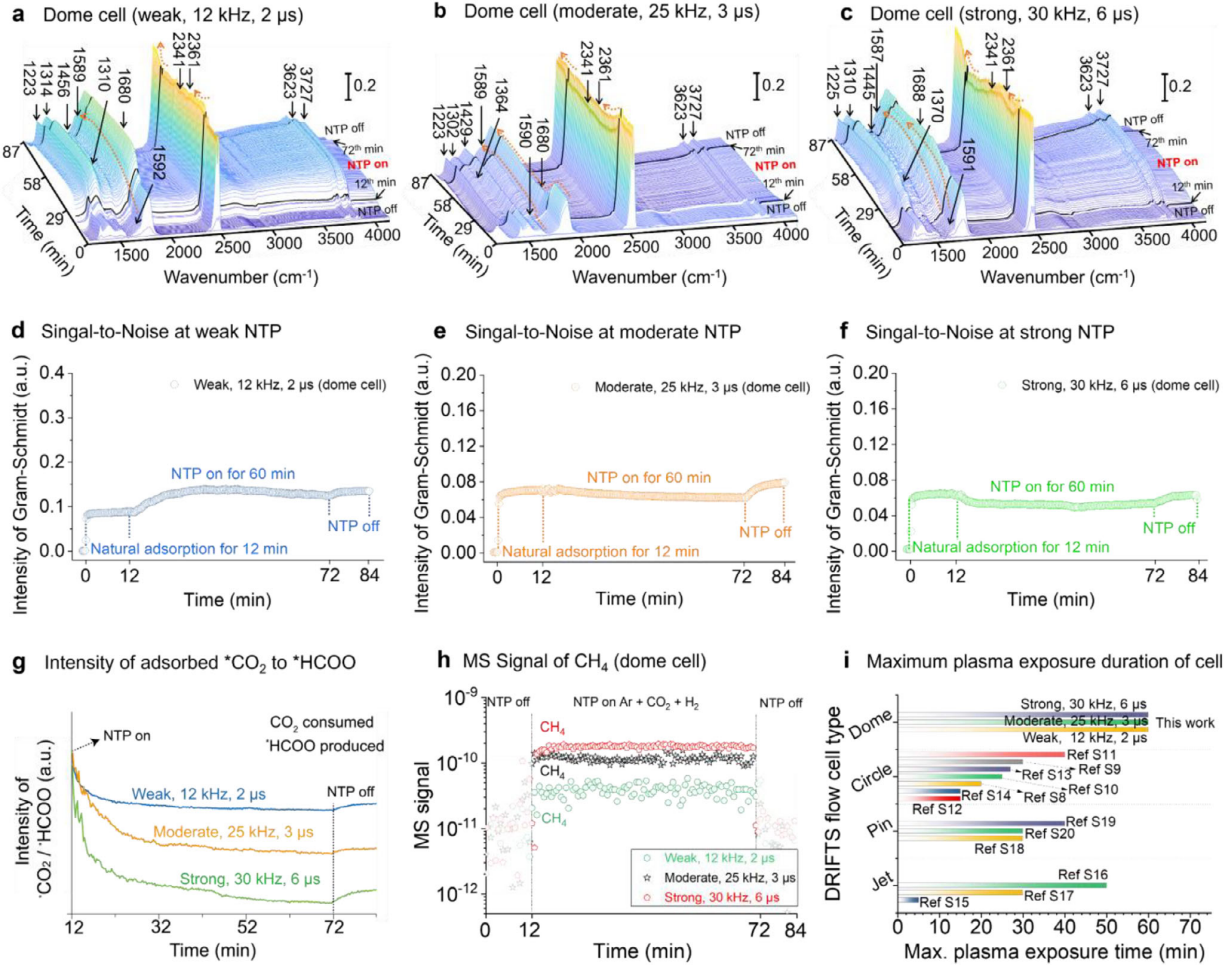
a–c) In situ DRIFTS spectra of CO_2_ hydrogenation over Ni/MgAlO_x_ in the dome cell under different pulse plasma discharge intensities (12 kHz, 2 µs; 25 kHz, 3 µs; 30 kHz, 6 µs); d–f) corresponding Gram–Schmidt intensity of the IR data (a–c); g) corresponding CO_2_/^*^HCOO intensity ratio; h) corresponding temporal MS signals (m/z = 15); i) Comparison of the reported plasma exposure time of different DRIFTS flow cells reported in this work and literature reports. (Refs. S8–S20 see Table S1, Supporting Information for details) (All experiments were performed using a feed gas of 95 vol% Ar + 1 vol% CO_2_ + 4 vol% H_2_ at 1 atm, pCO_2_ = 0.01 atm, pH_2_ = 0.04 atm; total flowrate = 50 mL min^−1^; bulk cell temperature during DRIFTS ≈54–60 °C).

The dome cell also enables reliable acquisition of in situ IR signals during plasma‐catalytic CO_2_ hydrogenation under steady‐state plasma‐on conditions for 60 min across different excitation regimes. Stable and distinct IR spectral responses of the catalyst surface were observed under weak, moderate, and strong glow intensities (**Figure** **3**a–c). In all cases, the spectra exhibited flat, drift‐free baselines, consistent with the stable discharge characteristics of the dome cell. The corresponding Gram‐Schmidt integrated IR intensities (Figure 3d–f) remained nearly constant throughout each 60 min plasma‐on period, with very low standard deviations (weak: ≈7.83 × 10^−3^; moderate: ≈4.54 × 10^−3^; strong: ≈4.88 × 10^−3^). These minimal fluctuations confirm that plasma instability, thermal perturbations, and instrumental noise are effectively suppressed, ensuring accurate in situ IR signal acquisition that faithfully represents surface reaction dynamics under various plasma conditions. By contrast, DRIFTS measurements performed under equivalent weak excitation (12 kHz, 2 µs) using jet, circle, and pin cells exhibited significant intensity fluctuations (standard deviation: jet ≈1.44 × 10^−2^; circle ≈2.74 × 10^−2^; pin ≈5.02 × 10^−2^; Figure S17a–c, Supporting Information). Such instability compromises spectral reliability and hampers quantitative peak analysis, thereby limiting the applicability of these configurations for long‐term in situ DRIFTS characterization of plasma catalytic systems.

The stable operation of the dome cell under varying plasma intensities enables a reliable mechanistic interpretation of plasma‐catalytic CO_2_ hydrogenation. Distinct spectral variations were observed in the IR spectra (Figure 3a–c). For example, the CO_2_/^*^HCOO signal ratio decreases progressively with increasing excitation intensity (Figure 3g, qualitative comparison). Since the bulk bed temperature remained nearly constant (≈54–60 °C), this behavior cannot be attributed to thermal effects. Instead, OES diagnostics (Figure S18, Supporting Information), conducted under representative weak, moderate, and strong excitation conditions, revealed a monotonic increase in *T*_e_ (from ≈1.7 to 2.5 eV) and *n*_e_ values (0.73–1.20 × 10^22^ cm^−3^), accompanied by a rising normalized Ar^749nm^/IR^631nm^ intensity (1.22 → 1.59 → 1.71; Figure S18, Supporting Information). These results confirm that the decline of the CO_2_/^*^HCOO ratio results from enhanced plasma reactivity rather than thermal effects. Specifically, elevated excitation intensity increased the generation of reactive species (such as hydrogen radicals and vibrationally excited CO_2_), which promoted the conversion of gas‐phase CO_2_ to ^*^HCOO intermediates via plasma‐assisted surface activation. Additionally, the IR intensities in Figure 3d–g remain largely unchanged upon plasma extinction, reflecting the slow relaxation kinetics of the surface‐adsorbed species and the excellent thermal stability of the dome cell, which prevents abrupt perturbations during plasma‐off transitions. Corresponding MS signals (Figure 3h; Figure S19a–c, Supporting Information) capture dynamic gas‐phase responses, showing progressive increases in the *m/z* = 15 (CH_4_) signal with increasing plasma intensity. This correlation between surface spectral evolution and bulk gas composition further confirms that the dome cell enables direct, synchronized observation of surface‐gas coupling phenomena in plasma catalysis. Collectively, these results demonstrate that the dome‐type DRIFTS cell maintains stable electrical and spectroscopic performance across a wide energy input window, enabling extended operation with high signal fidelity. The cell's exceptional discharge uniformity, thermal, and spectral stability make it a powerful diagnostic platform for in situ and *operando* investigations of plasma‐catalytic processes. In contrast, conventional DRIFTS cell designs exhibit limited operational lifetimes (typically 10–40 min; Figure 3i) due to discharge instability and thermally induced effects such as local overheating and IR baseline drift.

### 2.4 Dome DRIFTS Cell Reveals Further Mechanistic Information of Plasma Catalytic CO_2_ Methanation

**Figure 4:**
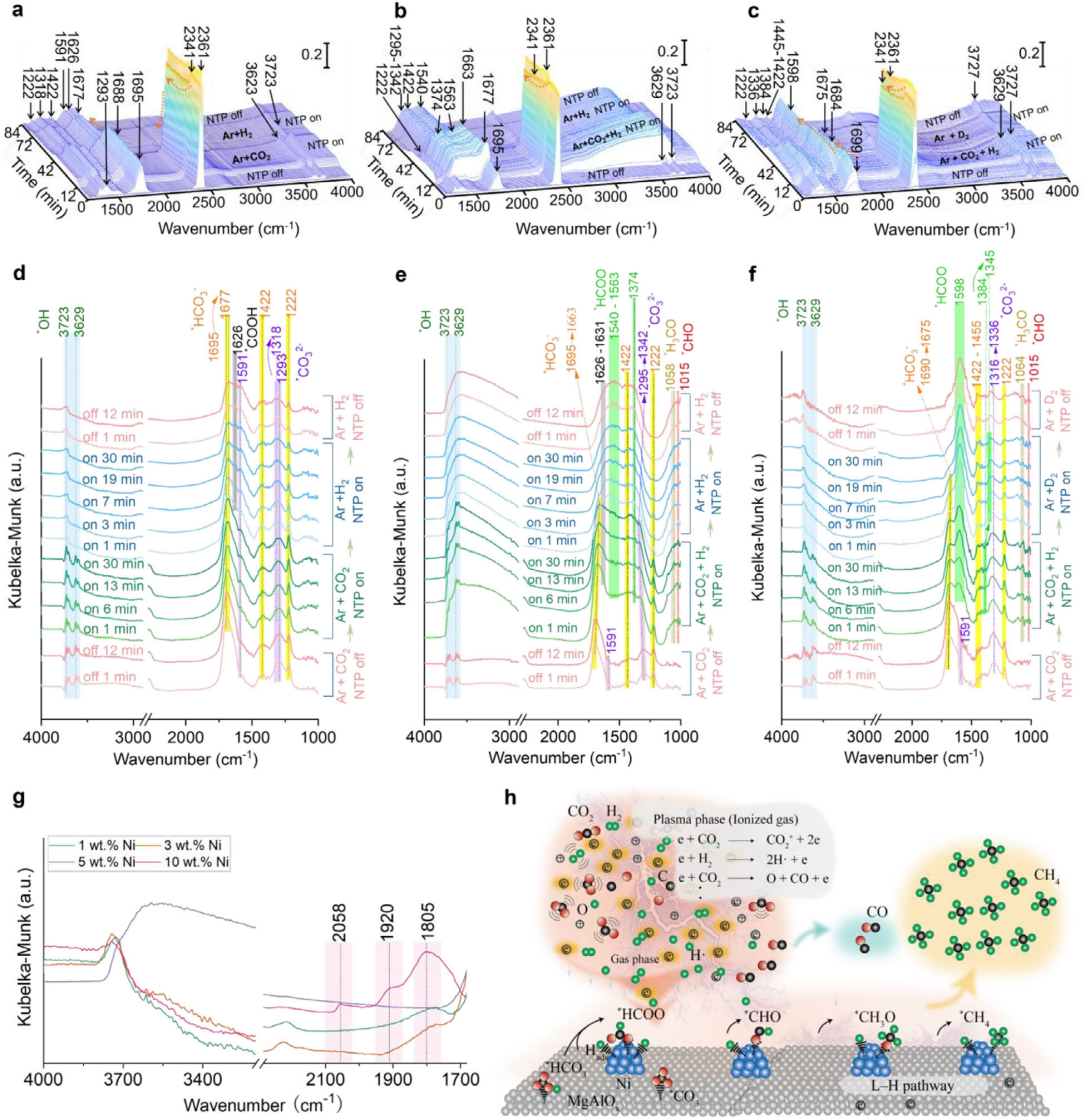
3D plots of the DRIFTS spectra of the dome cell in the transition‐state a) from 99 vol% Ar + 1 vol% CO_2_ NTP (pCO_2_ = 0.01 atm) to 96 vol% Ar + 4 vol% H_2_ NTP (pH_2_ = 0.04 atm), b) from 95 vol% Ar + 1 vol% CO_2_ + 4 vol% H_2_ NTP (pCO_2_ = 0.01 atm, pH_2_ = 0.04 atm) to 96 vol% Ar + 4 vol% H_2_ NTP (pH_2_ = 0.04 atm), and c) from 95 vol% Ar + 1 vol% CO_2_ + 4 vol% H_2_ NTP (pCO_2_ = 0.01 atm, pH_2_ = 0.04 atm) to 96 vol% Ar + 4 vol% D_2_ NTP (pD_2_ = 0.04 atm), over Ni/MgAlO_x_ catalyst. d–f) Corresponding detailed spectra showing time‐resolved evolution. g) Spectra under 96 vol% Ar + 4 vol% H_2_ NTP (pH_2_ = 0.04 atm) at 30^th^ min with different Ni loadings. h) Schematic illustration of the proposed reaction pathway. Experiments in (a, b) were conducted under 5 kV, 20 kHz, 1 µs pulse input; experiment in (c) under 5 kV, 30 kHz, 6 µs; atmospheric pressure; bulk cell temperature ≈54–60 °C.

The exceptional discharge stability and operational robustness of the dome‐type DRIFTS cell enable in situ experiments that were previously unattainable using conventional designs, particularly gas‐switching experiments essential for mechanistic elucidation. Accurate and reproducible acquisition of dynamic in situ IR spectra under realistic discharge environments, such as those resembling DBD operation, provides new opportunities to probe plasma‐induced surface processes with high temporal and chemical resolution. Here, using CO_2_ methanation over Ni/MgAlO_x_ as a model reaction, three gas‐switching experiments (**Figure** **4**; Figure S20, Supporting Information; corresponding OES results in Figure S21, Supporting Information) were proposed (I: NTP with CO_2_ → NTP with H_2_; II: NTP with CO_2_ + H_2_ → NTP with H_2_; III: NTP with CO_2_ + H_2_ → NTP with D_2_) to systematically explore the reaction mechanisms of plasma catalytic CO_2_ methanation under the pulse conditions. Control experiments were conducted to exclude contributions from the support or intrinsic surface groups. In detail: i) the bare MgAlO_x_ support showed no detectable intermediates under CO_2_, CO_2_ + H_2_, H_2_, CO_2_ + D_2_, and D_2_ atmospheres, either with or without plasma (Figure S22, Supporting Information); ii) Spectra recorded under pure Ar for both MgAlO_x_ and Ni/MgAlO_x_ (NTP off–on–off cycles, Figure S23) also revealed no discernible surface features. These results confirm that the IR bands observed in subsequent gas‐switching experiments originate exclusively from reactions occurring on Ni active sites (Note that CO_2_ conversions in these DRIFTS experiments were ≈6%, Note S2, Supporting Information).

In plasma catalysis, the coexistence of the L–H and E–R/L–R mechanisms^[^ ^10^, ^51^ ^]^ was hypothesized previously.^[^ ^3^, ^52^ ^]^ Density functional theory (DFT) calculations suggest that plasma‐activated species can significantly lower the activation barriers for E‐R reactions,^[^ ^8^, ^53^ ^]^ yet direct experimental evidence has been scarce. Leveraging the stability of the dome cell, Experiment I was designed to test whether the plasma‐induced excited hydrogen species (e.g., H⋅) could directly interact (i.e., either from the gas phase or via transient physisorption) with pre‐adsorbed CO_2_‐derived intermediates, thereby manifesting a non‐classical E–R/L–R pathway in plasma catalysis. Initially without plasma (Figure 4a,d; Figure S24, Supporting Information), IR bands corresponding to carbonate (1591, 1293 cm^−1^), bicarbonate (1695,1422, 1222 cm^−1^), and OH groups (3629, 3727 cm^−1^) are detected on the catalyst surface under CO_2_/Ar flow (full band assignments in Table S3 and Note S3, Supporting Information). Upon plasma ignition, the asymmetric O─C─O stretching band of bicarbonate (ν_as_(O─C─O), 1695 cm^−1^) exhibited a red shift to 1677 cm^−1^, indicative of bond polarization by free electrons in gas discharge. This shift suggests increased electron density on oxygen atoms, which weakens the C─O bond (as vibrational frequency, ν, is proportional to the square root of the bond force constant 𝑘, 𝜈∝𝑘^1/2^), thereby facilitating subsequent bond cleavage or hydrogenation. Concurrently, MS detected CO (m/z = 28) immediately after plasma ignition (Figure S20a, Supporting Information), yet no adsorbed CO features (1850–2100 cm^−1^) appeared in the IR spectra, suggesting that CO (from plasma‐induced CO_2_ dissociation) does not accumulate on the surface of 5 wt.% Ni/MgAlOx in Experiment I.

Upon switching the gas feed from CO_2_/Ar i to H_2_/Ar under plasma (Figure S25, Supporting Information), new IR band appeared at 1626 and 1181 cm^−1^, assignable to carboxyl (^*^COOH) intermediates,^[^ ^16^, ^54^ ^]^ while the 1677 cm^−1^ bicarbonate band decreased in intensity, partially, not completely, as in Experiment II (to be discussed below). This behavior suggests the formation of ^*^COOH species via partial hydrogenation of the pre‐adsorbed bicarbonates (^*^HCO_3_^−^ (ads) + H⋅ (gas or physisorbed) → ^*^COOH (ads) + OH^−^ (ads)).^[^ ^8^, ^53^ ^]^ No C─H stretching features of formate species were detected in the 2700–3000 cm^−1^ region (Figure S26, Supporting Information), confirming that ^*^HCOO intermediates did not form at this stage. These results provide direct spectroscopic evidence supporting the occurrence of a non‐classical E–R/L–R process, wherein plasma‐activated H· species transiently interact with surface‐adsorbed intermediates. Given that the surface is likely pre‐saturated with carbonaceous species under these conditions, H_2_ dissociation on Ni might be limited, reinforcing that ^*^COOH formation proceeds predominantly via non‐chemisorbed H· species. Consistent with this, CH_4_ was not detected by MS (Figure S20a, Supporting Information), suggesting that the E–R/L–R pathways alone are insufficient for full hydrogenation to methane under these conditions.

In Experiment II, when CO_2_/H_2_ (Ar as balance) were co‐fed under NTP‐off conditions, similar carbonate and bicarbonate adsorption features (to those in Experiment I) were observed (Figure 4b,e; Note S4, Supporting Information), confirming that, in the absence of plasma, H_2_ remains inert, and CO_2_ adsorption dominates. Switching on plasma in a CO_2_/H_2_/Ar stream mimics reaction conditions in DBD, i.e., simultaneous activation of CO_2_ and H_2_, which reveals the dynamics of intermediates’ conversion. Specifically, the bicarbonate band at 1695 cm^−1^ underwent a pronounced red shift to 1663 cm^−1^ rapidly decayed in intensity. Compared to the 1677 cm^−1^ band shift in Experiment I (CO_2_/Ar with plasma on), this further red shift indicates stronger polarization and weakening of the C─O bond, driven by concurrent plasma activation of both CO_2_ and H_2_. Upon subsequent removal of CO_2_ from the feed (with plasma maintained), the bicarbonate band disappeared completely, contrasting with Experiment I, where carbonate species persisted.

The difference suggests competitive adsorption of plasma‐induced species on the catalyst surface. In Experiment I (CO_2_/Ar plasma), the catalyst surface was saturated with CO_2_‐derived species, limiting H⋅ chemisorption and hindering L–H type reactions (after switching to H_2_/Ar plasma). Conversely, in Experiment II, (CO_2_/H_2_/Ar plasma), the coexistence of both reactants enabled plasma‐activated H⋅ to absorb and react with surface intermediates via the L–H mechanism, leading to CH_4_ formation (MS evidence in Figure S20b, Supporting Information). Upon removing CO_2_ from the feed, adsorption of gas‐phase H⋅ continued, facilitating hydrogenation of residual surface bicarbonates (ultimately leading to the complete disappearance of their IR signals at 1663, 1422, and 1222 cm^−1^) to form formate (1540–1563 and 1374 cm^−1^), CHO (1015 cm^−1^),^[^ ^55^ ^]^ and CH_3_O (1058 cm^−1^)^[^ ^56^ ^]^ species. Concurrently, CH_4_, CO, and H_2_O were detected by MS (Figure S18b, Supporting Information), and a strong water adsorption band appeared at 3629 cm^−1^ (in sharp contrast to Experiment I), further corroborating progressive surface hydrogenation. Overall, the results show that on Ni/MgAlO_x_, plasma‐generated H· must first adsorb on Ni sites to form surface‐bound H before it can efficiently take part in stepwise hydrogenation. The reaction mainly follows the L–H mechanism, with the non‐classical E–R/L–R route playing only a minor role. This was further confirmed by experiments employing different Ni loadings (Figures S27 and S28, Supporting Information).

To identify the rate‐determining step in the L–H mechanism, an H_2_/D_2_ isotopic transient experiment was performed (Figure 4c,f; Note S5, Supporting Information) to induce kinetic isotopic effects. Upon switching the feed gas from H_2_/Ar to D_2_/Ar under plasma‐on conditions, the bicarbonate band (1690–1675 cm^−1^) gradually vanished (Figure 4c,f), mirroring the behavior observed in Experiment II and confirming its conversion to formates (note that stronger plasma excitation was employed in Experiment III to enhance isotopic sensitivity and minimize spectral masking by intermediate accumulation noted previously). Importantly, the ν_s_(O─C─O) symmetric stretching of ^*^H(D)COO exhibited a characteristic red shift from 1384 to 1345 cm^−1^, confirming isotopic substitution of H by D. This shift reflects the weakened C─D bond, which lowers the vibrational frequency of the O─C─O mode, consistent with previously reported isotope effects.^[^ ^57^ ^]^ Simultaneously, the intensity of ^*^CH(D)O (1015 cm^−1^) and ^*^CH(D)_3_O (1058 cm^−1^) bands decreased significantly, further supporting the structural incorporation of deuterium into the intermediates. A complementary steady‐state isotopic transient kinetic analysis (SSITKA)‐type experiment was conducted by switching the feed from CO_2_/H_2_/Ar to CO_2_/D_2_/Ar under plasma‐on conditions (Figure S29, Supporting Information), while maintaining a constant CO_2_ partial pressure to preserve surface equilibrium. The formate‐related band again showed a red shift (from 1374 to 1341 cm^−1^), confirming isotopic substitution and providing direct evidence that the hydrogenation of surface formates is the kinetically relevant, likely rate‐determining step in the plasma catalytic CO_2_ methanation over Ni/MgAlO_x_.

In the CO‐feed control DRIFTS experiment (NTP on–off–on cycles; Figure S30, Supporting Information), only gas‐phase CO peaks were detected on the bare MgAlO_x_ support, whereas the 5 wt.% Ni/MgAlO_x_ catalyst exhibited distinct linearly adsorbed (2028 cm^‒1^) and bridged (1857, 1891, and 1929 cm^‒1^) CO bands. The intensities of these bands remained unchanged upon plasma switching, confirming that i) CO readily adsorbs on metallic Ni under ambient conditions, and ii) plasma exposure does not significantly alter its adsorption behavior. However, this externally supplied CO environment differs from the actual plasma conditions, in which CO species are generated in situ via plasma‐induced CO_2_ dissociation. During CO_2_ plasma operation (Experiment I), CO was detected by MS but not by IR, suggesting that CO_2_‐derived intermediates, particularly carbonates and bicarbonates, occupy the active Ni sites, thereby hindering CO adsorption. Moreover, in the absence of hydrogen species, the ^*^COOH → ^*^CO conversion pathway can be excluded, indicating that the observed CO arises primarily from gas‐phase CO_2_ dissociation rather than surface reactions. When H_2_ or D_2_ was introduced (Experiments II and III), no new CO‐related IR bands or isotopic shifts were observed, suggesting that CO does not accumulate or undergo measurable hydrogenation on the catalyst surface under plasma conditions. At higher Ni loading (10 wt.%; Figure 4g), CO adsorption bands became markedly stronger (2058, 1920, and 1805 cm^‒1^), consistent with enhanced CO stabilization on aggregated Ni domains. Nevertheless, both CO_2_ conversion and CH_4_ selectivity declined relative to the 5 wt.% Ni/MgAlO_x_ catalyst. The stronger CO binding at high Ni density likely inhibits H_2_ activation and impedes the sequential hydrogenation of CO_2_‐derived intermediates, thereby reducing overall methanation efficiency. Collectively, these findings indicate that CO acts as a spectator rather than a key surface intermediate in plasma‐catalytic CO_2_ methanation over Ni/MgAlO_x_. The dome‐type DRIFTS cell enables this mechanistic distinction, revealing that plasma‐driven CO_2_ methanation proceeds predominantly via a surface formate‐mediated L–H pathway leading to CH_4_ formation, while a secondary, gas‐phase CO route accounts for the minor CO by‐product (Figure 4h).

## 3 Conclusion

This work demonstrates the design and validation of a dome‐type DRIFTS flow cell capable of accurate in situ and *operando* infrared measurements under plasma conditions. The newly developed dome cell reproduces both the electrical and fluidic characteristics of DBD systems, while offering superior discharge stability and operational robustness across a wide range of plasma excitation conditions. These advancements address the limitations of conventional DRIFTS cell configurations, enabling reliable mechanistic studies of plasma–catalyst interactions. Using plasma‐catalytic CO_2_ methanation over a supported Ni/MgAlO_x_ catalyst as a model reaction, *operando* DRIFTS and gas‐switching experiments (a pulse discharge condition) revealed the mechanistic features of the specific system as: i) plasma‐activated H· radicals can transiently interact with preadsorbed bicarbonates to form *COOH intermediates, providing direct evidence of the E–R/L–R process; ii) the dominant reaction pathway follows the L–H mechanism, in which surface carbonates react with adsorbed hydrogen species to sequentially form surface formate, CHO, and CH_3_O intermediates responsible for continuous CH_4_ production; and iii) CO species formed through gas‐phase CO_2_ dissociation either leave the system as gaseous CO or adsorb on Ni as the spectator, rather than acting as a key surface intermediate. Furthermore, SSITKA (with H_2_/D_2_ isotope switch) confirmed that the hydrogenation of surface formates constitutes the kinetically relevant, and likely rate‐determining, step in the plasma‐catalytic methanation process. Overall, the dome‐type DRIFTS cell provides a robust and representative platform for mechanistic exploration of plasma‐catalytic systems, bridging the gap between in situ spectroscopy and practical reactor conditions. The resulting mechanistic insights from this cell can advance the fundamental understanding of plasma–surface interactions and offer valuable guidance for the rational design of plasma catalysts and reactors toward enhanced energy efficiency and performance in hybrid plasma‐catalytic technologies.

## 4 Experimental Section

### Cell Design and Manufacturing

Four DRIFTS cells, **dome**, **pin**, **jet**, and **circle** configurations, were designed using *SolidWorks* and subsequently fabricated in‐house. Detailed 3D schematics of each cell are shown in Figure S2a,c,e,g (Supporting Information). The dome cell consists of a polyether ether ketone (PEEK) base (60 × 60 × 30 mm) with a central quartz cup (inner diameter 6 mm, depth 12 mm) for catalyst loading. A stainless‐steel HV electrode (Ø1.5 mm), insulated by a sealed‐end quartz tube (wall thickness 0.5 mm), is positioned ≈4 mm beneath the catalyst surface. Gas inlet and outlet ports (Ø3 mm) are symmetrically integrated into the base: the inlet channels gas through a stainless‐steel dome (cavity Ø16 mm, height 23 mm), while the outlet extracts gas through the catalyst bed from the base of the quartz cup. The dome also functions as the grounded electrode and includes two ZnSe windows for IR transmission and a quartz observation window for visual inspection. In the pin cell, a 3 mm tungsten rod serves as the HV electrode, encased within a 1 mm‐thick quartz tube and positioned ≈3 mm above the catalyst surface. The enclosure is composed of PEEK, with a stainless‐steel ground electrode embedded ≈4 mm below the catalyst. The gas inlet and outlet geometries are identical to those of the dome cell. The jet cell employs a 3 mm tungsten HV electrode enclosed in a quartz tube (inner diameter 4 mm), which simultaneously serves as a gas inlet (50 mL min^−1^) and discharge channel. The electrode tip is positioned ≈3 mm above the catalyst. The grounded electrode and PEEK dome enclosure follow the same configuration as in the pin cell. In the circle cell, the HV electrode is a stainless‐steel rod embedded ≈4 mm into the catalyst bed, while the ground electrode is a 1 mm copper wire coiled around the outer wall of the quartz cup. The dome enclosure fabricated from PEEK, and the gas flow path mirrors that of the dome cell. All flow cells were assembled using vacuum‐compatible fittings and heat‐resistant gaskets and are fully compatible with commercial DRIFTS accessories (e.g., Harrick Praying Mantis).

### Simulation Methods

Electrostatic field simulations were performed using Ansoft Maxwell V14.0 to evaluate the spatial distribution of the electric field across different DRIFTS cell geometries and a representative DBD reactor under an applied potential of 7 kV. The reduced electric field (E/N) was obtained by dividing the simulated electric field intensity by the gas number density at ambient pressure and temperature.

Complementary computational fluid dynamics (CFD) simulations were carried out in ANSYS Fluent employing a pressure‐based steady‐state solver coupled with the realizable *k–ε* turbulence model to characterize gas‐flow behavior within the same geometries. The computational domains were identical to those used in the electrostatic simulations to ensure consistency. The inlet boundary was defined by a mass flow rate of 50 mL min^−1^ with a feed composition of Ar/CO_2_/H_2_ = 95:4:1 (vol%), while the outlet was treated as a pressure boundary. All solid surfaces were modeled as no‐slip walls, and the catalyst bed was represented as a porous medium with a porosity of 0.4. Pressure–velocity coupling was implemented using the Coupled scheme.

### Methods for Plasma Discharge Diagnostics and Catalytic Performance Evaluation in the Dome Cell

To evaluate discharge properties of the DRIFTS cells under investigation, two types of power supplies were used including a sinusoidal power supply (CTP‐2000K, Nanjing Suman Plasma Technology Co., Ltd., China) for continuous sine‐wave excitation and a pulse power supply (MPP04‐A10A‐30, Kurita Co. Ltd., Japan) for generating both single‐pulse and multi‐pulse discharges^[^ ^40^ ^]^ (schematics of the plasma systems are shown in Figure S14a, Supporting Information). High‐speed imaging of the discharge in the cells was captured using a Canon 90D camera with an exposure time of 1/800 with plasma generated from pulse power at 5 kV, 20 kHz, and 1 µs. Simultaneously, the applied voltage and current were continuously monitored using an HV probe (P6015A, Tektronix) and a current monitor (CT‐E0.5, Bergoz), both connected to an oscilloscope (TBS1102C, Tektronix) for real‐time data acquisition. The bulk temperature of the DRIFTS cells during operation was measured using a FLIR TG165‐X infrared camera, while the plasma OES spectra were recorded using a Maya2000Pro spectrometer (Ocean Optics) to analyze excited species and plasma stability. Catalytic evaluation under DBD conditions was referenced from the previous study,^[^ ^40^ ^]^ where identical power supplies (sine and pulse), electrode configurations, GC protocols, and calculation methods for CO_2_ conversion, product selectivity, and energy efficiency were employed (see setup in Figure S14b, Supporting Information). The CH_4_ space‐time yield (STY, mmol/(*g*_cat_ × h)) was determined according to Equation 1.

(1) $$STY=\frac{F_{\mathrm{CH}4,\;\mathrm{out}}\times 10^{3}}{W}$$

where *F*_CH4,out_ (mol h^−1^) represents the molar flow rate of the CH_4_, W the weight of the packing catalyst (g).

For the dome cell, the catalytic performance was evaluated using a pulse power supply. ≈30 mg of the 5 wt.% Ni/MgAlO_x_ catalyst in powder form was loaded, and the reaction was carried out with a gas mixture of 1.6 vol% CO_2_/6.4 vol% H_2_/Ar at a total flow rate of 155 mL min^−1^, with all other details consistent with the aforementioned methods.

DRIFTS‐MS analysis was performed using a Nicolet iS50 FTIR spectrometer (Thermo Fisher Scientific) equipped with a Harrick Scientific Praying Mantis diffuse reflectance accessory, which was adapted to accommodate the custom‐designed in situ DRIFTS reaction cells. The outlet gas was simultaneously monitored using an analytical quadrupole mass spectrometer (QGA, Hiden Analytical, UK).

DRIFTS spectra were recorded with 32 scans at a resolution of 8 cm^−1^. Before the experiment, the pre‐reduced catalyst was treated in situ under a 10 vol% H_2_/Ar gas mixture (total flow rate at 50 mL min^−1^) at either sine (8.5 kV, 7.5 kHz) or pulse (5 kV, 20 kHz, 1 µs) plasma conditions for 10 min. Subsequently, the system was purged with pure Ar (50 mL min^−1^) for 5 min with the plasma switched off (NTP‐off) to remove physically adsorbed H_2_. The background spectrum was then recorded under the same pure Ar flow with sine or pulse excitation. DRIFTS spectra were processed and reported in Kubelka–Munk (KM) units, as this transformation minimizes wavelength‐dependent scattering effects and allows semi‐quantitative comparison of band intensities. DRIFTS experiments were conducted at a constant total flow rate of 50 mL min^−1^ with different gas compositions and under different plasma conditions (specific conditions can be found in the manuscript). Real‐time MS analysis was performed simultaneously with in situ DRIFTS experiments to monitor the outlet gas composition. The MS signals were tracked at m/z = 44 (CO_2_), 2 (H_2_), 18 (H_2_O), 28 (CO), 15 (CH_4_), 4 (D_2_), and 20 (CD_4_). The specific experimental procedure of the gas‐switching experiments is: i) natural adsorption for 12 min under the corresponding gas atmosphere without plasma (NTP‐off); ii) switching on plasma (NTP‐on) in the same gas atmosphere of (i) for 30 min; 3) switching to a new gas atmosphere under the NTP‐on condition for 30 min; and iv) switching off plasma (NTP‐off) for the final 12 min.

### Statistical Analysis

*Pre‐Processing*: DRIFTS spectra were converted to Kubelka–Munk units without any additional smoothing or filtering unless explicitly stated. Gram–Schmidt integrals were computed directly from raw spectra. OES data were averaged over three consecutive acquisitions for each experimental condition. GC‐derived conversions were recorded after 30 min of steady‐state operation, using three consecutive injections per condition. IR camera‐based temperature measurements represent the average of three readings.

*Data Presentation*: All quantitative results are reported as mean ± standard deviation (SD). Exact sample sizes (*n*) are specified in the main text and/or figure captions.

*Statistical Methods*: No formal hypothesis testing was conducted, as the study focuses on *operando* diagnostics and mechanistic interpretation rather than population‐based inference. Owing to the small number of technical replicates (typically *n* = 3), statistical analyses were limited to descriptive metrics (mean and SD) to assess reproducibility and signal stability, for example, the low SD observed in Gram–Schmidt integrals. Normality and variance homogeneity were not evaluated.

*Software*: Data processing was carried out in Microsoft Excel (2024). All figures, regression analyses, and statistical summaries were produced in OriginPro 2023b (OriginLab).

## Conflict of Interest

The authors declare no conflict of interest. Patents and patent applications related to the design and application of the dome DRIFTS flow cell described in this manuscript have been filed by Zhejiang Tihe Instrument Co., Ltd. with the China National Intellectual Property Administration (CNIPA) under the following application numbers of 202411562818.X and 20242268188.X (under patentability assessment), and 202422681881.X and 202422681882.4 (granted). Trademark (FANNE) registration application for the flow cell: No. 82746888 (applied on 24/12/2024 to CNIPA).

## Supporting information



Supporting Information

## Data Availability

The data that support the findings of this study are available from the corresponding author upon reasonable request.
