# Supplementary material for: Development of a Robust Diffuse Reflectance Infrared Fourier Transform Spectroscopy (DRIFTS) Cell for Elucidating Reaction Mechanisms in Nonthermal Plasma Catalysis
Source: Small Methods. 2025 Nov 20;9(12):e01403. doi: 10.1002/smtd.202501403 (PMC12716174; doi:10.1002/smtd.202501403)
Supplement: Supplementary file 1 — Supporting Information [file SMTD-9-e01403-s001.docx]

Supporting Information

Development of a Robust Diffuse Reflectance Infrared Fourier Transform Spectroscopy (DRIFTS) Cell for Elucidating Reaction Mechanisms in Nonthermal Plasma Catalysis

Jiangqi Niu*, Shaowei Chen, Yi Chen, Jinyan Zhang, Guanting Zhou, Huiji Yu, Qingyang Lin, Tianqi Liu, Shanshan Xu, Zifu Li, Jianguo Huang, Huanhao Chen*, Xiaolei Fan*

Table of Contents

Supplementary Notes

Supplementary Tables

Supplementary Figures

Supplementary References

**Supplementary Note 1**

**Determination of Electron Temperature Using Argon Emission Line Ratios**

The electron temperature (*T_e_*) in dielectric barrier discharge (DBD) systems containing argon can be estimated using the intensity ratio of specific argon emission lines^1,2^. For this purpose, 10 mL/min of Ar was deliberately added to the reaction mixture (10 mL/min CO_2_ + 40 mL/min H_2_) during separate DBD experiments for plasma diagnostics (note: Ar was not used in the subsequent catalytic tests). The emission lines at 750.4 nm and 811.5 nm are particularly suitable due to their sensitivity to electron collisions. The intensity ratio of these lines is directly related to the electron excitation rate coefficient (*k_e_*), enabling the calculation of *T_e_*.

The emission intensity of the respective lines is expressed as:

$I_{750.4}=C_{750.4}h\nu_{750.4}A_{ij}^{750.4}\frac{n_{e}k_{e}^{750.4}n_{Ar}}{A_{i}^{750.4}+k_{q}^{750.4}n_{Ar}}$ (Eq. S1)

$I_{811.5}=C_{811.5}h\nu_{811.5}A_{ij}^{811.5}\frac{n_{e}k_{e}^{811.5}n_{Ar}}{A_{i}^{811.5}+k_{q}^{811.5}n_{Ar}}$ (Eq. S2)

where:

$C$ is a constant incorporating optical and geometric factors,

$h\nu$ is the photon energy $h\nu=hc/\lambda$ (with Planck's constant $h$, speed of light $c$, and wavelength $\lambda$),

$A_{ij}$ is the transition probability,

$n_{e}$ and $n_{Ar}$ represent electron and argon densities, respectively,

$k_{e}$ is the electron excitation rate coefficient,

$A_{i}$ corresponds to the total transition probability, defined as $A_{i}=1/\tau_{0}$, where $\tau_{0}$ is the natural lifetime,

$k_{q}$ is the quenching rate coefficient for the excited argon state.

The density of argon atoms $n_{Ar}$ can be estimated using the ideal gas law:

$n_{Ar}=\frac{p}{kT_{g}}$ (Eq. S3)

where the pressure $p$ is set to 1.01 × 10⁵ Pa and $T_{g}$ is the gas temperature.

The electron temperature $T_{e}$ is determined by solving the empirical equation that describes the relationship between the electron excitation rate coefficients:

$\frac{k_{e}^{811.5}}{k_{e}^{750.4}}=0.99e^{-T_{e}/4.69}+2.31e^{-T_{e}/0.43}+6.27e^{-T_{e}/0.03}+0.45$ (Eq. S4)

By substituting the measured intensity ratio into this equation (**Table S2**), $T_{e}$ can be obtained.

**Determination of Electron Density via Stark Broadening of Argon Lines**

The electron density $n_{e}$ can be determined using Stark broadening, a method similar to that employed for hydrogen lines^3^. In the case of argon, the emission line at 696.54 nm is particularly useful for this analysis. The electron density is calculated as:

$n_{e}=\left(\frac{\text{Δ}\lambda_{Stark}}{2\times10^{-11}}\right)^{3/2}$ (Eq. S5)

**Supplementary Note 2**

**Catalyst Preparation**

The Ni-based catalysts were synthesized according to our previous work^4^. In a typical synthesis, a calculated amount of nickel nitrate hexahydrate (Ni(NO_3_)_2_·6H_2_O, Chron Chemicals, 98 %) and 0.95 g of Hydrotalcite (Mg_4_Al_2_(OH)_12_CO_3_·3H_2_O, Macklin, AR) were individually dissolved in 10 mL and 20 mL of anhydrous ethanol. The amount of Ni(NO_3_)_2_·6H_2_O was adjusted to achieve the target Ni loadings (1, 3, 5, 10 wt.%) relative to the total mass of the catalyst. Subsequently, the Ni precursor solution was gradually introduced into the hydrotalcite suspension under vigorous stirring. The suspension was continuously stirred in a water bath (maintained at 80 °C) until the solution evaporated completely (about 1 hour). The solid obtained was collected in a crucible and calcined under static air at 450 °C for 2 h (ramping rate 5 °C/min), yielding the Ni/MgAlO_x_ catalyst with different Ni loadings. Prior to DBD or DRIFTS experiments, the catalyst was further reduced in a 40 vol% H_2_/60 vol% Ar atmosphere (flow rate of 50 mL/min) at 450 °C for 2 h.

**Catalyst Characterization**

XRD patterns were collected using a SmartLab diffractometer (Rigaku Corporation, Japan) with Cu-*K*α radiation in the 2θ range of 10° to 80° (with a scan speed of 10°/min) at 40 kV and 30 mA. H_2_/CO_2_-TPD and H_2_ pulse chemisorption measurements were performed on a fully automated instrument (BELCAT II, Japan) equipped with a thermal conductivity detector (TCD). In H_2_/CO_2_-TPD, before H_2_/CO_2_ adsorption, 30 mg of the as-prepared catalyst was reduced in a 10% vol% H_2_/Ar gaseous mixture stream at 450 °C for 2 h, then cooled down to 50 °C under pure Ar flow. Subsequently, H_2_/CO_2_ adsorption was carried out at 60 °C by flowing a 10 vol% H_2_ or CO_2_ (balanced with He) gas mixture for 1 hour. After that, the sample was purged with pure He for 1 hour to remove physically adsorbed H_2_/CO_2_. Finally, the temperature-programmed desorption of H_2_/CO_2_ was recorded in a He flow by ramping the temperature from 60 to 750 °C at a ramp rate of 10 °C/min. In case of H_2_ pulse chemisorption, the as-prepared catalyst (0.1g) underwent pre-treatment at 300 °C under Ar flow. The gas was later replaced with H_2_ and heated to 450 °C at a heating speed of 5 ℃/min. After 2 h, the gas was again exchanged with Ar and purged until cooling to ambient temperature. Lastly, pulsing with H_2_ was carried out until adsorption saturation. Based on the saturated adsorption amount and each adsorption amount, calculate the chemical adsorption amount of hydrogen to obtain the metal dispersion. The microscopic features of the Ni/MgAlO_x_ catalyst were investigated via high-resolution transmission electron microscopy (HRTEM) using a Talos F200S G2 instrument (operated at an accelerating voltage of 200 kV, Thermo Fisher Scientific, America). To investigate the atomic dispersion of nickel within the catalyst, aberration-corrected high-angle annular dark-field scanning transmission electron microscopy (HAADF-STEM) was performed using a Spectra 300 dual aberration-corrected microscope (Thermo Fisher Scientific, America) operated at an accelerating voltage of 300 kV with a beam current of 50 pA. In addition, energy-dispersive X-ray spectroscopy (EDS) mapping was carried out to examine the spatial distribution of Ni, Al, Mg, and O elements in the catalyst. For sample preparation, a small amount of catalyst powder was ultrasonically dispersed in anhydrous ethanol for 5 min to form a homogeneous suspension. A few drops of this suspension were then carefully deposited onto an ultra-thin copper grid and dried before transmission electron microscopic analyses. The actual metal loading of the as-prepared catalyst was determined using the inductively coupled plasma optical emission spectrometer (ICP–OES, PE Avio 200), and the sample was dissolved with a potent acid.

**Structural Characterization and Catalytic Performance of 5 wt.% Ni/MgAlO_x_ in Plasma CO_2_ Methanation**

The X-ray diffraction (XRD) pattern of the reduced 5 wt% Ni/MgAlO_x_ catalyst (**Figure S11**) shows diffraction peaks corresponding to MgAl_2_O_4_ (spinel phase, 35–37°) and MgO (43° and 60°)^4^. Conversely, diffraction peaks of Ni^0^/NiO were not detected, indicating the likely presence of small Ni species on the support, which was confirmed by high-resolution transmission electron microscopy (HRTEM) characterization. The layered double hydroxide (LDH)-derived MgAlO_x_ has a sheet-like morphology (**Figures S12**), benefiting the dispersion of Ni species (Ni nanoparticles of 5–10 nm on the support). HAADF-STEM micrographs show that Ni nanoparticles are primarily distributed along the boundary of MgAlO_x_, and such a configuration might benefit plasma catalytic CO_2_ hydrogenation, as it maximizes active site accessibility. Ni/MgAlO_x_ (reduced) has surface basic sites potentially favoring the interaction with CO_2_ during the catalysis, as evidenced by CO_2_-temperature programmed desorption (CO_2_-TPD) characterization (**Figure S13a**) showing two desorption peaks at approximately 154 °C and 596 °C, indicating the presence of different types of surface basic sites^5^. The desorption peak at ~154 °C corresponds to weak basic sites, typically associated with the weakly bound bicarbonate and surface carbonate species^6^. The desorption peak at ~596 °C represents strongly bound carbonate species^7^. These strong basic sites may play a role in CO_2_ activation by stabilizing intermediates such as carbonate or formate species, thereby facilitating CO_2_ hydrogenation under plasma conditions^8^. We assessed Ni/MgAlO_x_ for NTP catalytic CO_2_ methanation using a DBD rig (**Figure S14**) under both pulse and sine plasma excitation, employing 330 mg of catalyst in pellet form (30–40 mesh). The results (**Figure S15**) show the catalyst performed better in pulse plasma than sine plasma, e.g., CH_4_ selectivity: 97.7% (pulse) vs. 94.7% (sine). The CO_2_ conversion in the dome-type DRIFTS cell was around 6 %, representing a low-conversion regime suitable for mechanistic investigation, compared with 72.1 % (sine) and 75.3 % (pulse) in the DBD reactor.

**Supplementary Note 3**

In plasma catalytic systems, previous studies have hypothesized that the plasma-induced vibrationally excited and/or dissociative species could enable the Eley–Rideal (E–R) mechanisms in addition to the common Langmuir–Hinshelwood (L–H) mechanisms. For instance, Xu et al.^9^ suggested that gas-phase H radicals may participate in surface hydrogenation via the E–R route, while Kim et al.^10^ speculated on similar possibilities but acknowledged the lack of direct experimental support. Findings of recent DFT studies also suggest the presence of the E–R pathways in plasma catalysis. Nozaki et al.^11^ assume the preferential adsorption of the plasma-activated H atoms on the catalyst surface, which can react with the gas-phase CO_2_ with much lower activation energy than the reaction with the surface-bound CO_2_. Annemie et al.^12^ showed that plasma species notably reduce the activation barrier for formate formation via E–R-like pathways. Yet, to date, no direct in situ experimental evidence has been reported to verify the occurrence of E–R reactions in plasma catalysis.

To explore this possibility, a gas-switching experiment (CO_2_ + Ar → Ar + H_2_, the experiment I in the manuscript) was conducted to probe the initial surface reactions of CO_2_ under plasma conditions. Under NTP-off conditions, DRIFTS spectra revealed the rapid formation of bidentate carbonate (1591, 1293 cm⁻^1^), bicarbonate (1695, 1422, 1222 cm⁻^1^), and CO_2_-induced OH species (3629, 3727 cm⁻^1^), showing the surface species variation due to CO_2_ adsorption on the catalyst surface sites. Upon plasma ignition, the bicarbonate band at 1695 cm⁻^1^ exhibited a distinct red shift to 1677 cm⁻^1^, suggesting electronic perturbation of the surface intermediates. Simultaneously, CO formation was immediately detected in the gas phase by MS (m/z = 28), while no IR signatures of adsorbed CO species (1850–2100 cm⁻^1^) were observed (likely the pre-ignition saturation of the surface sites prevents CO adsorption under CO_2_ plasma).

Subsequently, when switching to an Ar + H_2_ atmosphere under plasma, a new band at 1626 cm⁻^1^ emerged, potentially corresponding to carboxyl (^*^COOH) species^13^ formed via interaction between gas-phase H species and surface bicarbonate. The activation energy for the surface reaction H· + HCO_3_⁻ → ^*^COOH + OH⁻ is estimated to be approximately 50–70 kJ/mol, consistent with typical energy barriers (40–80 kJ/mol) reported for plasma-activated hydrogen species reacting with adsorbed CO_2_-derived intermediates^11,12^. Meanwhile, the CO_2_-induced OH band at 3629 cm⁻^1^ disappeared, while the stable OH band at 3727 cm⁻^1^ remained. No CH_4_ production was detected by MS during experiment I.

**Supplementary Note 4**

This gas-switching experiment (CO_2_ + H_2_ + Ar → H_2_ + Ar, under constant plasma excitation, experiment II in the manuscript) was conducted to investigate the formation and evolution of surface intermediates during CO_2_ hydrogenation under steady-state plasma conditions. Under NTP-off conditions, bidentate carbonate (1591, 1295 cm⁻^1^), bicarbonate (1695, 1422, 1222 cm⁻^1^), and OH groups (3629, 3727 cm⁻^1^) were immediately observed on the catalyst surface. Plasma ignition led to a significant red shift of bicarbonate bands (1695 → 1663 cm⁻^1^). New surface species including formate (*HCOO, 1540–1563 and 1374 cm⁻^1^), *CHO (1015 cm⁻^1^), and *CH_3_O (1058 cm⁻^1^) were formed under plasma conditions. MS simultaneously detected CH_4_, CO, and H_2_O as the products.

After switching off CO_2_ (Ar + H_2_ plasma), the bidentate carbonate bands disappeared completely, while *HCOO, *CHO, and *CH_3_O species gradually weakened but remained detectable. During experiment II, the details of formation/consumption of surface intermediates were recorded, confirming the major involvement of bicarbonate species in the CO_2_ methanation in the pulse plasma discharge.

**Supplementary Note 5**

This gas-switching experiment (CO_2_ + H_2_ + Ar → D_2_ + Ar, under plasma excitation, experiment III in the manuscript) was designed to probe the incorporation of hydrogen into surface intermediates via isotopic substitution, thereby identifying the kinetically relevant hydrogenation steps in the reaction mechanism. Under NTP-off conditions, DRIFTS spectra showed the formation of bidentate carbonate (1591, 1316 cm⁻^1^), bicarbonate (1690, 1422–1445, 1222 cm⁻^1^), and OH groups (3629, 3727 cm⁻^1^). Plasma activation caused a red shift in bicarbonate bands (1690 → 1675 cm⁻^1^) and the disappearance of the 1422–1445 and 1222 cm⁻^1^ bands. Surface formate (*HCOO, 1598 cm⁻^1^), *CHO (1015 cm⁻^1^), and *CH_3_O (1058 cm⁻^1^) species were detected.

After switching to Ar + D_2_ plasma, the bidentate carbonate bands were suppressed, while bicarbonate and OH bands significantly decreased. The HCOO⁻ red-shifted from 1384 cm⁻^1^ to 1345 cm⁻^1^. CHO and CH_3_O peaks also weakened under D_2_, reflecting isotope substitution effects. A weak CO adsorption peak appeared at 2105 cm⁻^1^, which is attributed to isotopic substitution effects at the catalyst surface. Specifically, this ^*^CO signal likely originates from deuterium replacing pre-adsorbed hydrogen species that had partially occupied active sites prior to D_2_ introduction. The kinetic isotope effect slows down subsequent hydrogenation steps, resulting in the transient accumulation of CO. In contrast, during experiment I, where ^*^COOH was generated via gas-phase H attack on surface ^*^HCO_3_⁻, no CO formation was observed, implying that even H-mediated conversion to ^*^CO is kinetically hindered. Therefore, under D_2_ flow, CO formation is further suppressed.

**Supplementary Tables**

**Table S1.** Comparison of the relevant state-of-the-art plasma DRIFTS studies.

| Reaction | Electrical parameters | Feed gas | Catalyst | Plasma exposure time (min) | Cell type and refs. |
| --- | --- | --- | --- | --- | --- |
| Water–gas shift | Generator: sine  Voltage: 4–6 kV  Frequency: 22.5 kHz | 0.7 vol% CO  2.8 vol% H_2_O/Ar  Total flow = 150 mL/min | HKUST–1 | - | circle^14^ |
| CO_2_ hydrogenation to CH_4_ | Generator: sine  Voltage: 6 kV  Frequency: 26 kHz | 4 vol% CO_2_/16 vol% H_2_ /Ar  Total flow = 75 mL/min | Ni/BETA | 20 | circle^15^ |
|  | Generator: sine  Voltage: 5 kV  Frequency: 23 kHz | 4 vol% CO_2_/16 vol% H_2_ /Ar  Total flow = 75 mL/min | Ni/UIO–66 | 30 | circle^16^ |
|  | Generator: sine  Voltage: 5 kV  Frequency: 23.5 kHz | 1 vol% CO_2_/4 vol% H_2_/Ar | Ru/MgAlO_x_ | 25 | circle^17^ |
| CH_4_ oxidation | Generator: sine  Voltage: 5 or 6 kV  Frequency: 27 kHz | 0.5 vol% CH_4_/10 vol% O_2_  0.5 vol% Kr/Ar  Total flow = 100 mL/min | Pd/Al_2_O_3_ | 40 | circle^18^ |
| VOCs decomposition | Generator: sine  Voltage: 14 kV  Frequency: 2 kHz | 400 ppm toluene  20 vol% O_2_/N_2_  Total flow = 30 mL/min | SrTiO_3_ | 15 | circle^19^ |
| Dehydration of crystalline hydrates | Generator: sine  Voltage: 0.9 kV  Frequency: 27 kHz | Air or Ar | KBr | 27 | circle^20^ |
| CO_2_ hydrogenation to CH_3_OH | Generator: sine  Voltage: 24 kV  Frequency: 9.5 kHz  Discharge power: 14.4 W | 25 vol% CO_2_/75 vol% H_2_  Total flow = 40 mL/min | Fe_2_O_3_/  γ–Al_2_O_3_ | 15 | circle^21^ |
| Hydrocarbon selective catalytic reduction deNO_x_ | Generator: sine  Voltage: 4–7.5 kV  Frequency: 20 kHz | 720 ppm NO/4.3 vol% O_2_  4340 ppm C_1_/4 vol% H_2_O  0.3 vol% Kr/He  Total flow = 100 mL/min  Plasma jet: 50 mL/min Ar | Ag/Al_2_O_3_ | 5 | jet^22^ |
| CH_4_  decomposition | Generator: pulse  Frequency: 14.3 MHz  Discharge power: 2 W | 0.5 vol% CH_4_/Ar  Total flow = 402 mL/min  Plasma jet: 200 mL/min Ar | Ni powder | 50 | jet^23^ |
| CO_2_ hydrogenation to CH_3_OH | Generator: sine | H_2_ + CO_2_ gas stream | MnO_x_/ZrO_2_ | 30 | jet^24^ |
| VOCs decomposition | Frequency: 500 Hz  Voltage: 16 kV  Discharge power:  24 mW | Total flow = 30 mL/min | γ–Al_2_O_3_  TiO_2_  CeO_2_ | 30 | pin^25^ |
| CH_4_  dry reforming | Generator: sine  Voltage: 5.5 kV  Frequency: 50 Hz | 10 mL/min CO_2_ or CH_4_  and 60 or 100 mL/min He | La–Ni/Al_2_O_3_  Ni/Al_2_O_3_ | 40 | pin^26^ |
| CO_2_ hydrogenation to CH_4_ | Generator: pulse  Voltage: 8 kV  Frequency: 1 kHz  Pulse length: 50 µs | 20 vol% CO_2_/80 vol% H_2_  Total flow = 12.5 mL/min | Co/CeZrO_4_ | 30 | pin^27^ |

**Table S2**. Spectral data for Ar emission lines (NIST Database)

| Wavelength  (nm) | Photon Energy  (J) | Transition Probability  $A_{ij}$ (s^–1^) | Total Transition Probability  $A_{i}$ (s^–1^) | Lifetime  $\tau_{0}$ (ns) |
| --- | --- | --- | --- | --- |
| 750.4 | 2.64 × 10^–19^ | 4.45 × 10^7^ | 4.69 × 10^7^ | 21.3 |
| 811.5 | 2.45 × 10^–19^ | 3.31 × 10^7^ | 3.31 × 10^7^ | 30.2 |

**Table S3.** IR Band assignments of the adsorbed species identified for plasma catalytic CO_2_ hydrogenation under ambient conditions.

| Surface species | Assignment | Wavenumber (cm^–1^) | |
| --- | --- | --- | --- |
|  |  | This work | Literature value |
| Bidentate carbonate  b–^*^CO_3_^2–^ | υ_as_ (O–C–O) | 1596 (**Figure 2**)  1591, 1592 (**Figure 3**)  1591 (**Figure 4**) | 1598^28^  1624–1697^29^  1570–1580^30^ |
|  | υ_s_ (O–C–O) | 1357 (**Figure 2**)  1314, 1302, 1310 (**Figure 3**)  1293–1318 (**Figure 4a**)  1294–1342 (**Figure 4b**)  1316–1336 (**Figure 4c**) | 1325^30^ |
| Bicarbonate  ^*^HCO_3_^–^ | υ_as_ (O–C–O) | 1677 (**Figure 2**)  1680, 1688 (**Figure 3**)  1695, 1688, 1677 (**Figure 4a**)  1699, 1677, 1663 (**Figure 4b**)  1699, 1684, 1675 (**Figure 4c**) | 1654, 1676, 1685^31^ |
|  | υ_s_ (O–C–O) | 1438 (**Figure 2**)  1456, 1429, 1445 (**Figure 3**)  1422 (**Figures 4a** and **b**)  1422–1455 (**Figure 4c**) | 1409^32^  1417^33^  1433, 1443, 1439^28^  1412^34^  1430^30^  1335^35^ |
|  | δ (O–H) | 1222 (**Figures 2, 4**)  1223, 1225 (**Figure 3**) | 1242^33^  1273^32^  1227, 1231^28^  1224^34^  1200–1225^30^ |
| Bidentate formate  b–^*^HCOO | υ_as_ (O–C–O) | 1566 (**Figure 2**)  1589, 1587 (**Figure 3**)  1540–1563 (**Figure 4b**)  1598 (**Figure 4c**) | 1587^36^  1572, 1570^28^  2876, 2961^35^  1561^31^  1572, 1570^28^  1580^33^  1575–1595^30^ |
|  | υ_s_ (O–C–O) | 1374 (**Figure 2**)  1371, 1364, 1370 (**Figure 3**)  1374 (**Figure 4b**)  1384–1345 (**Figure 4c**) | 1387, 1382^28^  1360–1370^30^ |
| ^*^CHO | / | 1015 (**Figure 4c**) | 1017^37^ |
| Methoxy  ^*^H_3_CO | υ_s_ (C–O) | 1058 (**Figure 4b**)  1064 (**Figure 4c**) | 1056^34^  1040^38^  1050–1060^30^ |
| Absorbed linear CO  ^*^CO | υ_as_ (C–O) | 2105 (**Figure 4c**) | 2078^39^ |
| Bi-bridged OH group | –O–H | 3623 (**Figures 2, 3** and **4**) | 3612, 3627, 3654^40^ |
| Tridentate OH group | –O–H | 3727 (**Figures 2, 3** and **4**) | 3702, 3735^40^ |

**Table S4**. Physicochemical properties of the Ni/MgAlO_x_ catalyst with different Ni loading amounts.

| Catalyst | Uptake (mmol/g_cat_) | | Relative coverages (%) | | Actual loading^a^ (wt.%) | Metal dispersion^b^ (%) |
| --- | --- | --- | --- | --- | --- | --- |
|  | H_2_ | CO_2_ | H_2_ | CO_2_ |  |  |
| 1 wt.% Ni | 0.22 | 0.90 | 5.0 | 95.0 | 1.4 | 65.2 |
| 3 wt.% Ni | 0.12 | 0.78 | 3.5 | 96.5 | 3.3 | 47.9 |
| 5 wt.% Ni | 0.10 | 0.83 | 6.1 | 93.9 | 5.6 | 37.7 |
| 10 wt.% Ni | 0.05 | 0.83 | 5.9 | 94.1 | 11.8 | 24.2 |

^a^Ni loading of the as-prepared catalysts, measured by ICP-OES; ^b^Metal dispersion of the as-prepared catalysts, measured by H_2_ pulse chemisorption.

To quantitatively assess the relative coverage of H_2_ and CO_2_ on the metal surfaces of catalysts under investigation, H_2_/CO_2_-TPD experiments were conducted. The absolute uptake of H_2_ and CO_2_ (mmol/g_cat_) was quantified from the corresponding TPD profiles. Given that the bulk gas temperature in NTP catalysis typically does not exceed 200 °C, the analysis was deliberately focused on the weakly and moderately adsorbed species by integrating the TPD signals only up to this temperature. This approach provides a more realistic estimation of the adsorbate populations under actual plasma operation conditions, as strongly bound species desorbing at higher temperatures are less likely to be involved in the plasma-catalytic process. The relative surface coverage was defined as the molar percentage of each gas, calculated from its individual uptake (mmol/g_cat_, ≤ 200 °C) relative to the total uptake of both gases (mmol/g_cat_, ≤ 200 °C). This metric enables a direct comparison of the abundance of the two key reactants on the catalyst surface.

**Supplementary Figures**


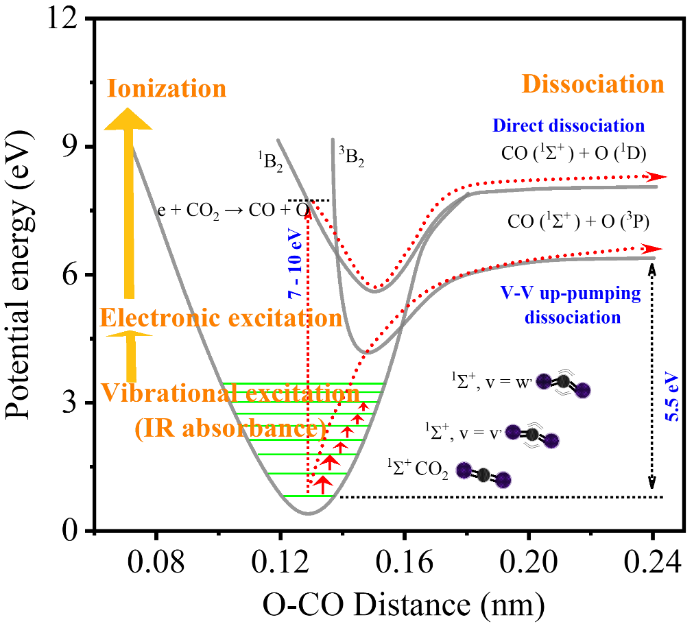


**Figure S1**. Schematic of CO_2_ electronic and vibrational levels, including direct electronic excitation-dissociation, stepwise vibrational excitation.


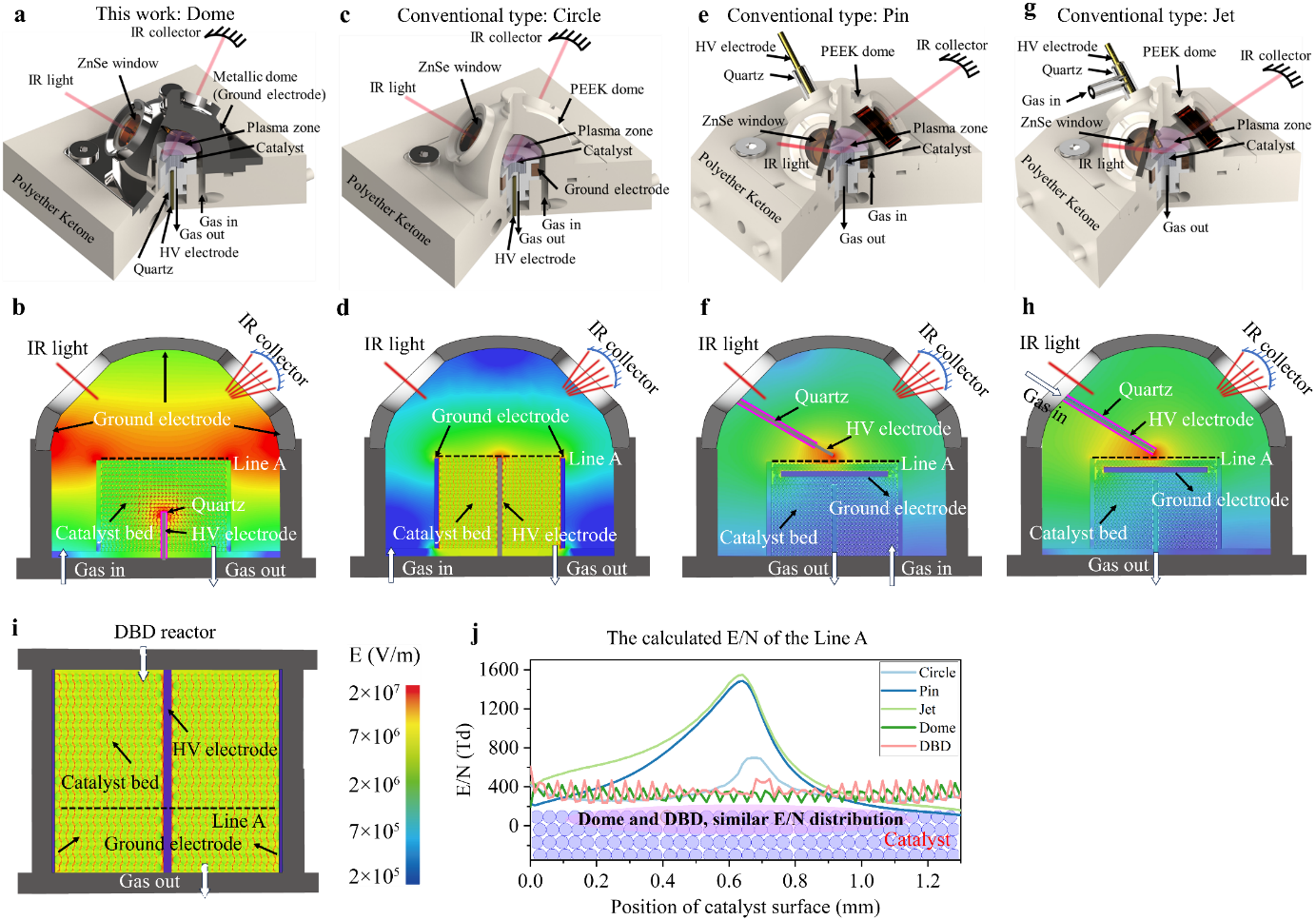


**Figure S2**. Schematics and simulated electric field distributions of various plasma cells, including (a, b) dome cell (this work), (c, d) conventional circle cell, (e, f) pin cell, and (g, h) jet cell. (i) Electric field distribution in a typical DBD reactor. (j) Comparison of the calculated reduced electric field strength along Line A (the dashed lines in b, d, f, h, i) above the surface of catalyst beds in DRFITS cells and across the catalyst bed in a DBD reactor.


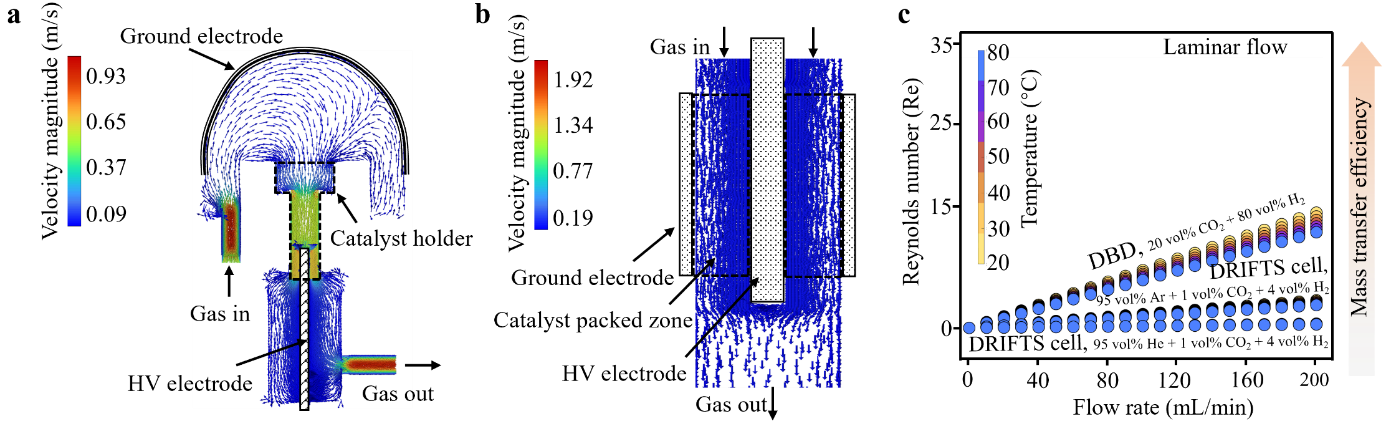


**Figure S3**. (a, b) Simulated flow field distributions (by CFD) in the dome DRIFTS cell and DBD reactor at a gas flow rate of 90 mL/min. (c) Flow regime analysis of the DRIFTS cell and DBD reactor as a function of flow rate and temperature.


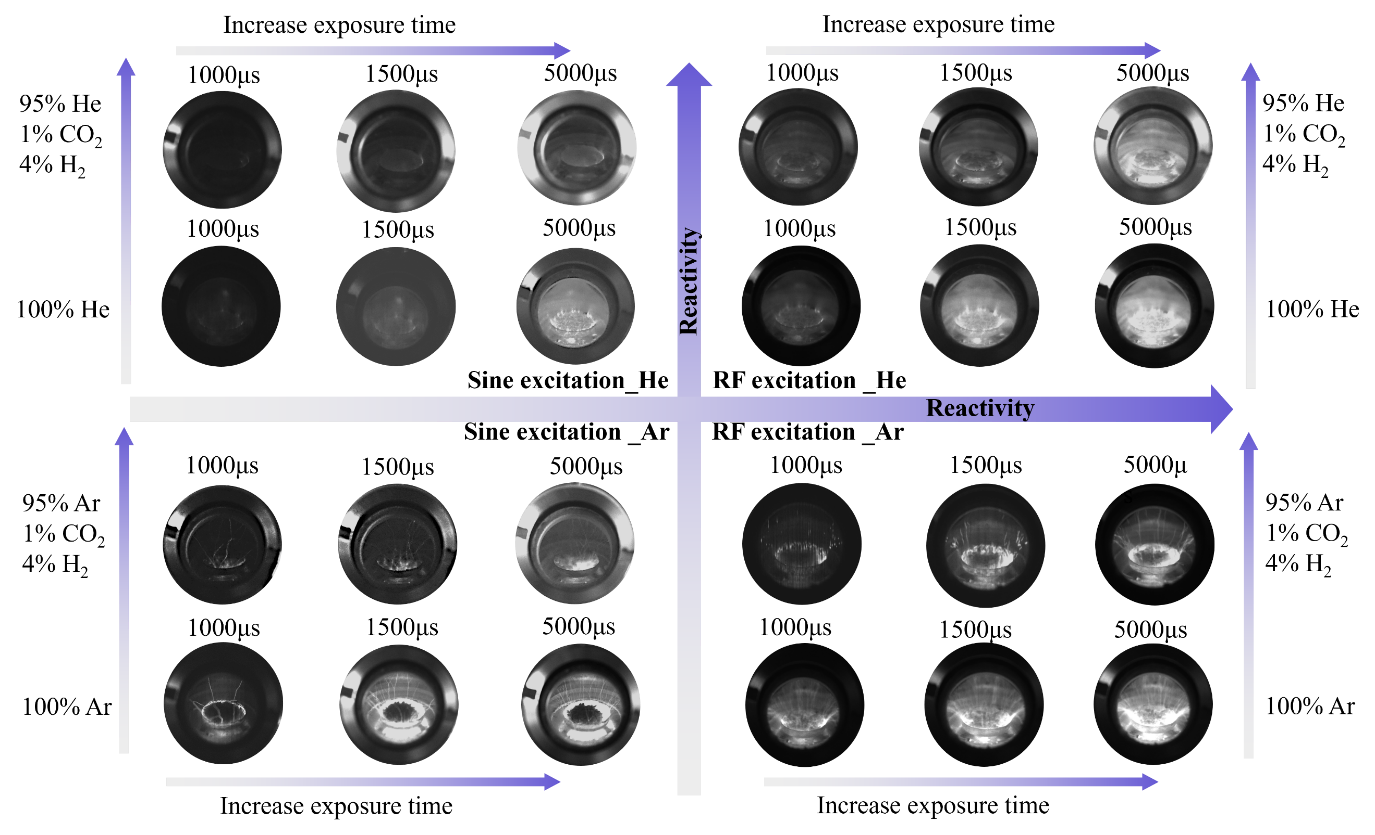


**Figure S4**. Discharge morphologies of the dome DRIFTS cell under sine and pulse excitation in different gas atmospheres (Ar, He, Ar+CO_2_+H_2_, He+CO_2_+H_2_). Images were recorded using a digital camera with exposure times of 1000 μs, 1500 μs, and 5000 μs, under otherwise identical settings, to capture the discharge morphology clearly under different plasma intensities.


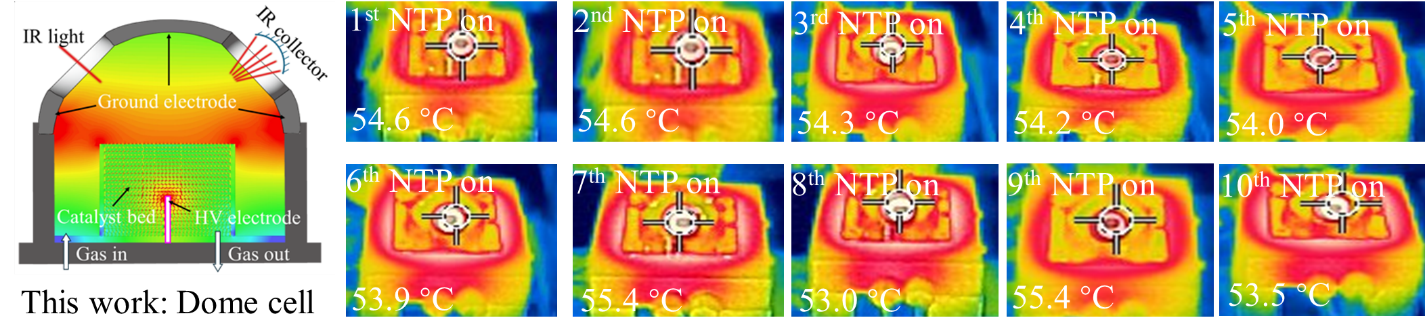


**Figure S5**. Temperature variation during the NTP on-off cycles (pulse excitation at 5 kV, 20 kHz, 1 μs) of the dome cell.


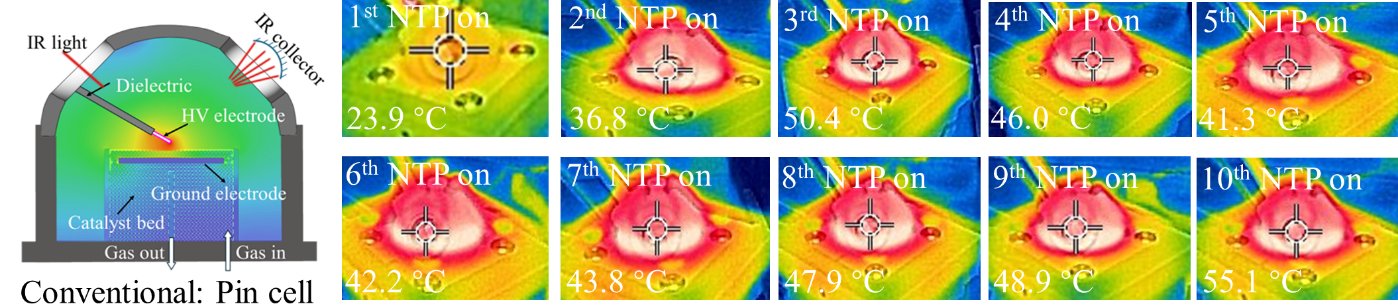


**Figure S6**. Temperature variation during the NTP on-off cycles (pulse excitation at 5 kV, 20 kHz, 1 μs) of the conventional pin cell.


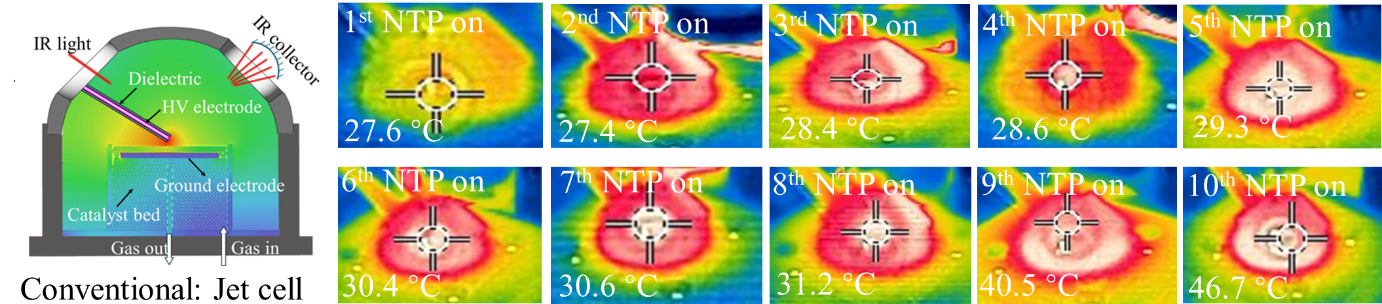


**Figure S7**. Temperature variation during the NTP on-off cycles (pulse excitation at 5 kV, 20 kHz, 1 μs) of the conventional jet cell.


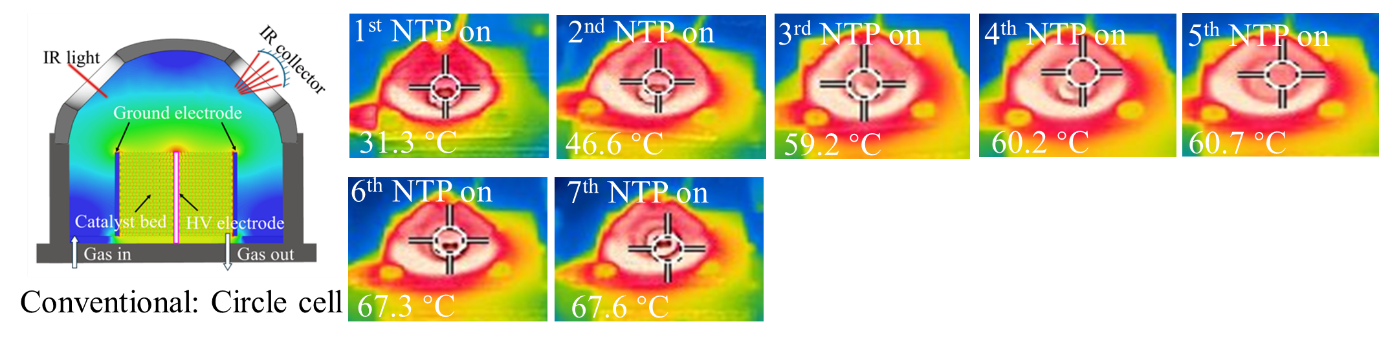


**Figure S8**. Temperature variation during the NTP on-off cycles (pulse excitation at 5 kV, 20 kHz, 1 μs) of the conventional circle cell.


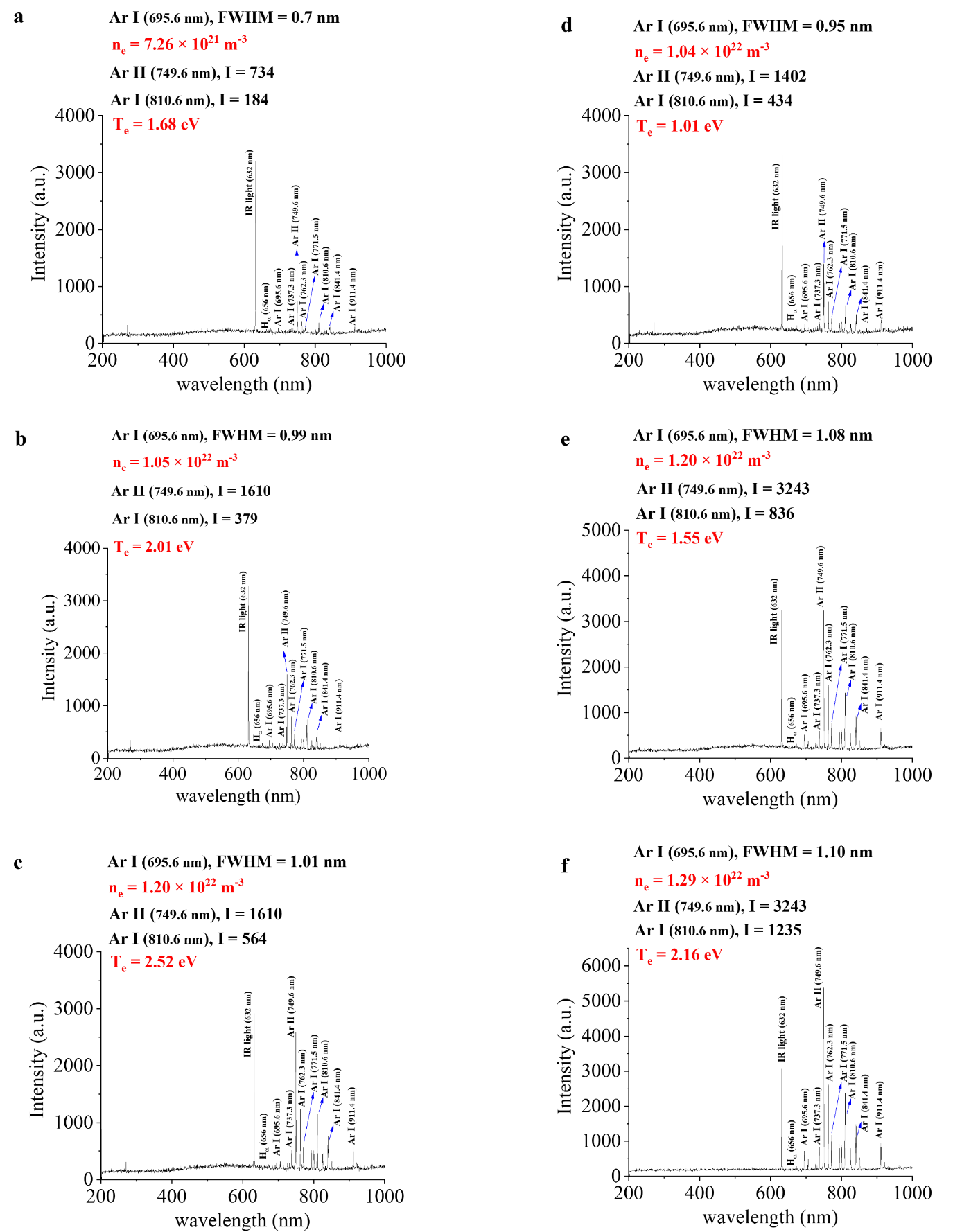


**Figure S9.** OES spectra collected from the dome cell in Ar (45 mL/min) + CO_2_ (1 mL/min) +H_2_ (4 mL/min) under pulse (5 kV, 1 μs) of (a) 10 kHz, (b) 20 kHz, (c) 30 kHz, and sine (7.5 kHz) of (d) 8.5 kV, (e) 9.5 kV, (f) 10.5 kV.


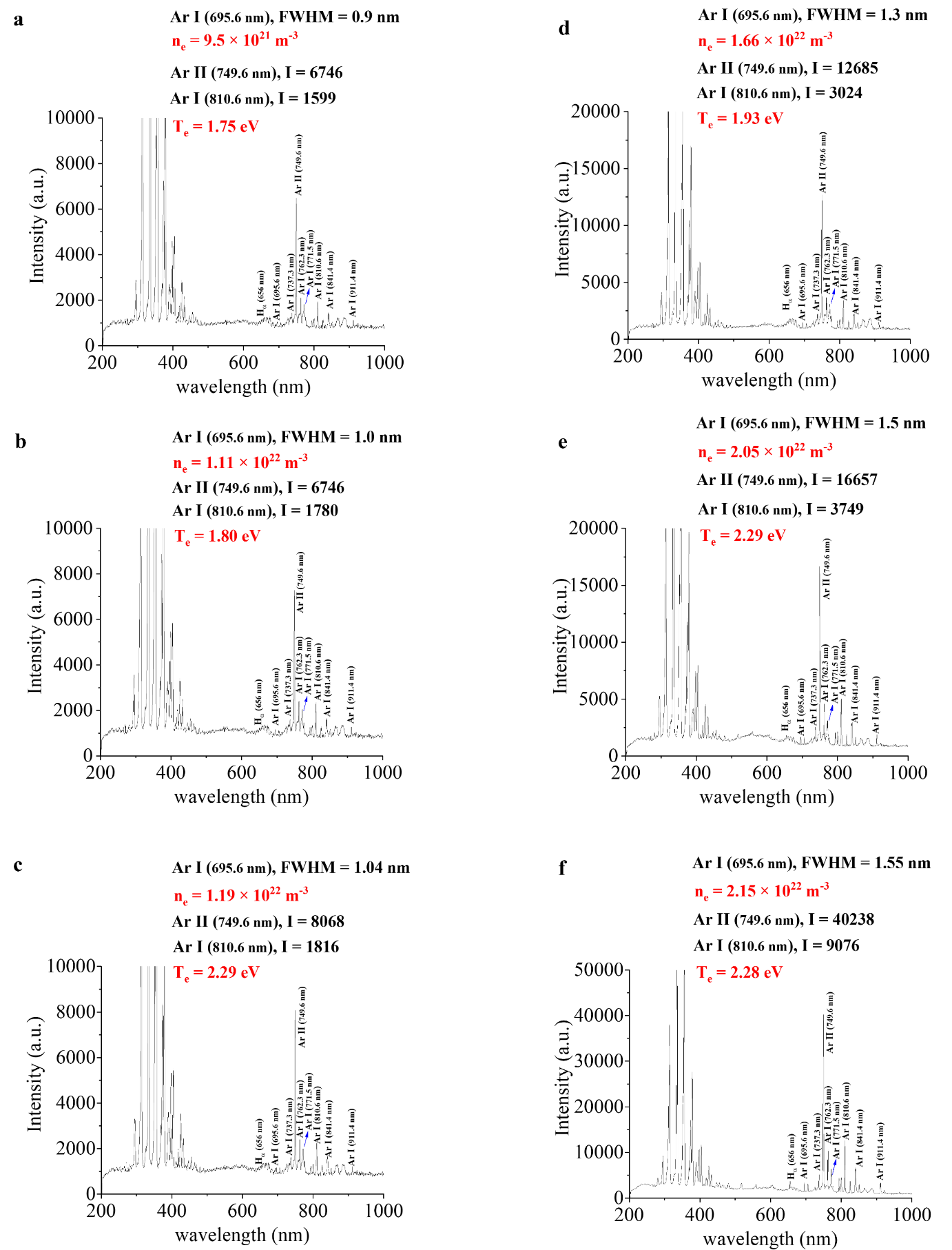


**Figure S10.** OES spectra collected from DBD in Ar (10 mL/min) + CO_2_ (10 mL/min) +H_2_ (40 mL/min) under pulse (5 kV, 1 μs) of (a) 10 kHz, (b) 20 kHz, (c) 30 kHz, and sine (7.5 kHz) of (d) 8.5 kV, (e) 9.5 kV, (f) 10.5 kV.


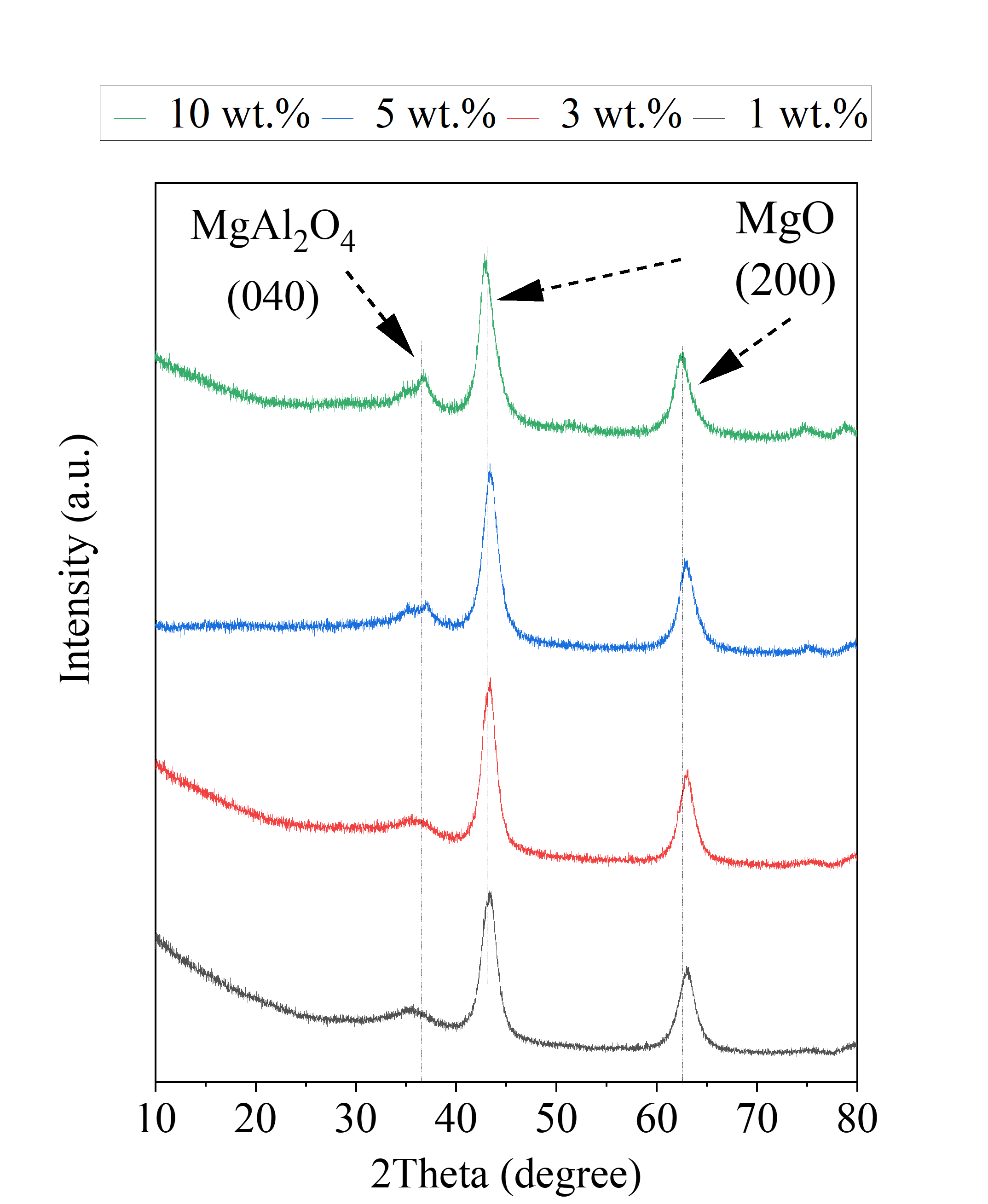


**Figure S11.** XRD patterns of the reduced Ni/MgAlO_x_ catalyst with different Ni loadings.


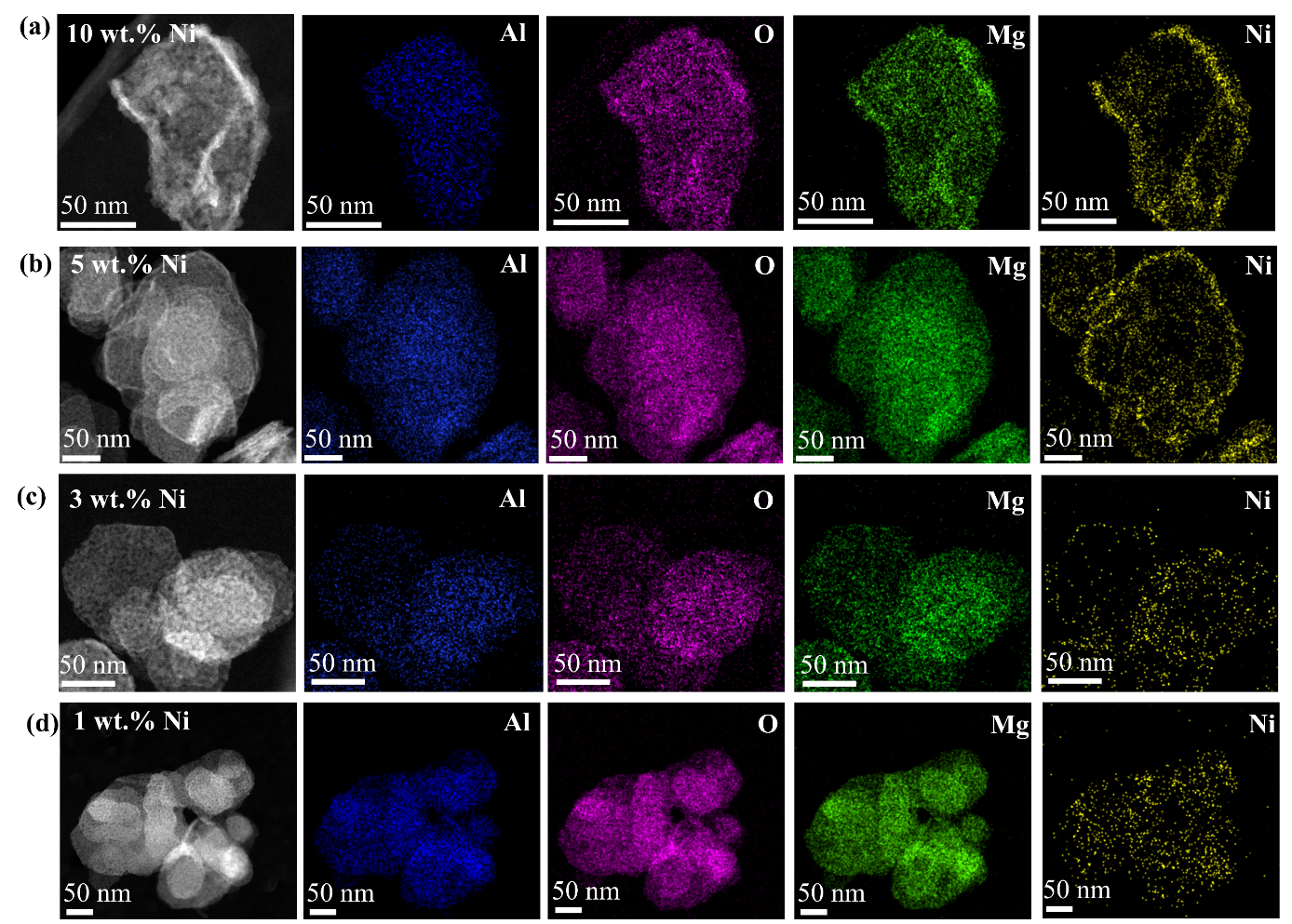


**Figure S12.** (a-d) HAADF-STEM image and the corresponding EDXS elemental maps of the Ni/MgAlO_x_ catalyst with different Ni loadings.


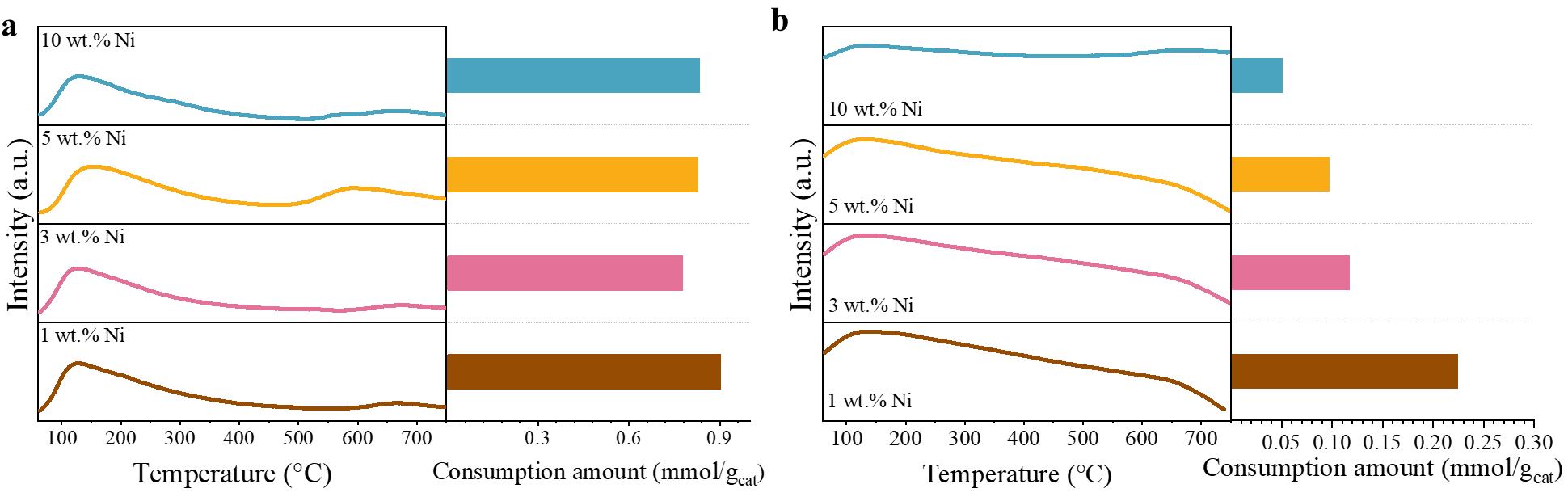


**Figure S13.** (a) CO_2_-TPD and (b) H_2_-TPD profiles of the Ni/MgAlO_x_ catalyst with different Ni loadings.


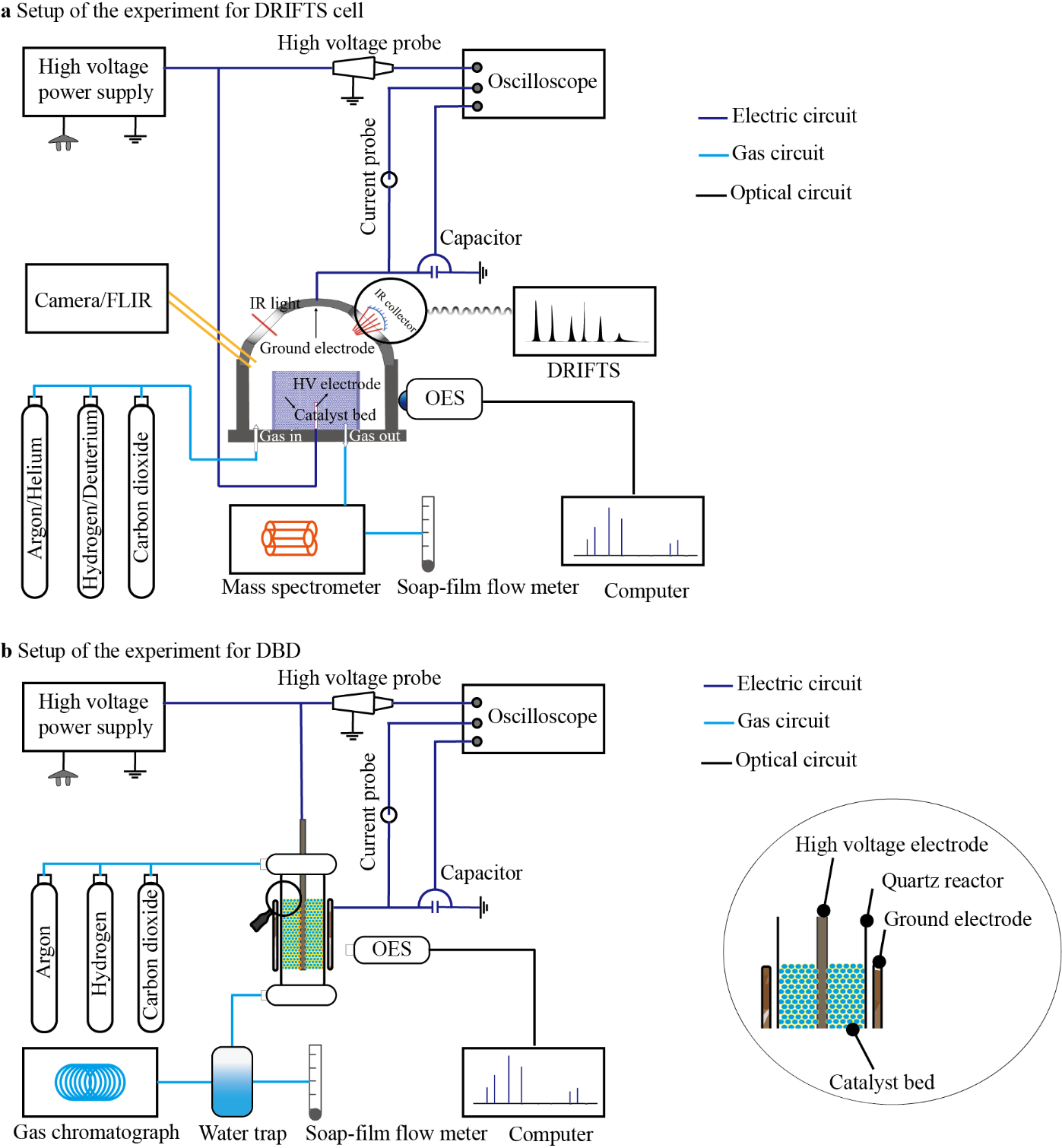


**Figure S14**. The experimental rig for (a) operando DRIFTS-OES-MS experiments and (b) DBD plasma catalytic CO_2_ hydrogenation.


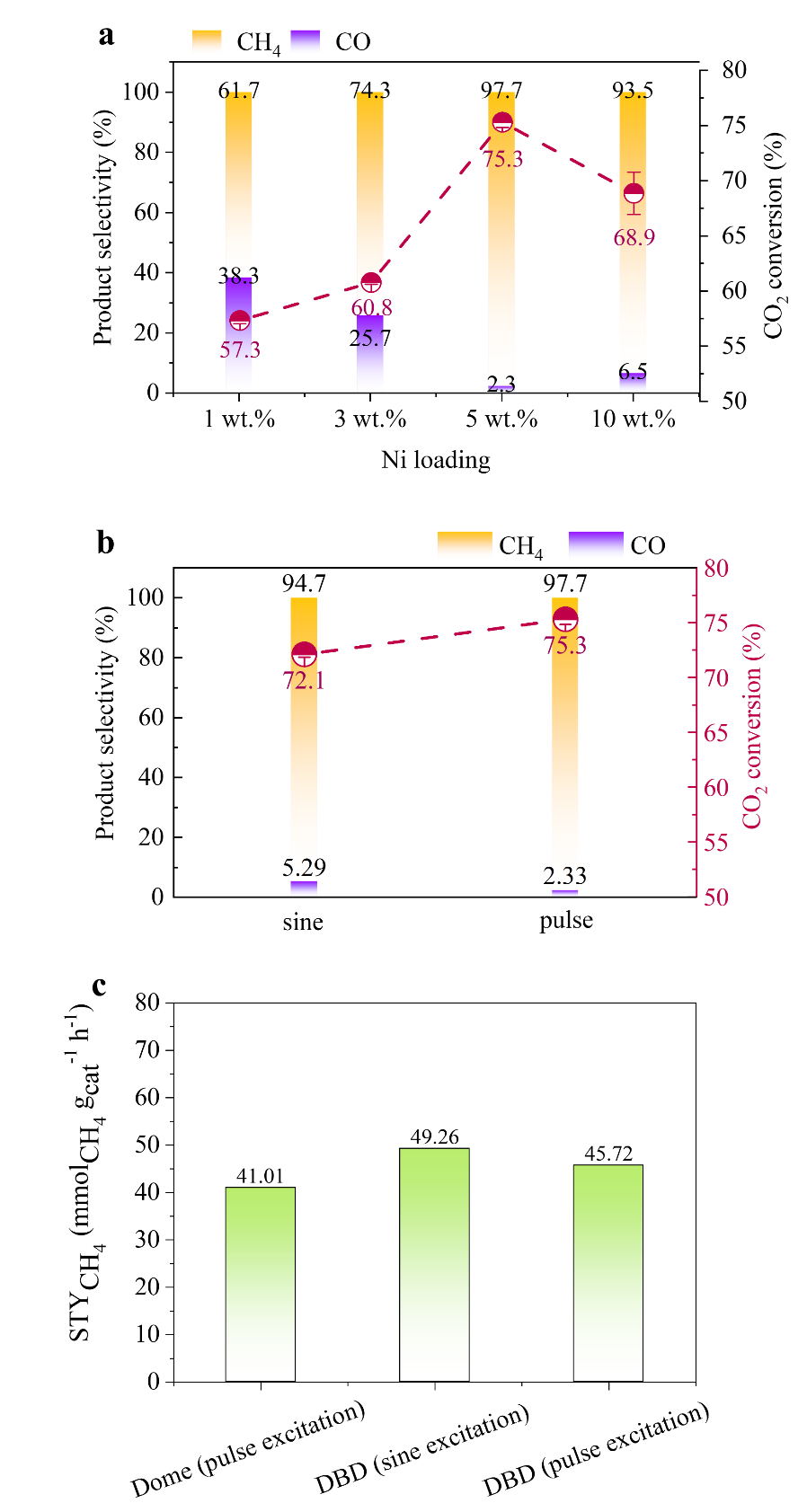


**Figure S15.** (a) Performance of plasma catalytic CO_2_ methanation under DBD with sine excitation of Ni with different loading amounts (1, 3, 5, 10 wt.%); (b) comparison of CO_2_ methanation of 5 wt% Ni under sine or pulse excitation; (c) STY of dome, DBD under sine or pulse excitation. (DBD: ∼400 mg Ni/MgAlO_x_, 500–600 μm, 30 mm discharge length, 80 vol% H_2_/20 vol% CO_2_, 50 mL min⁻^1^ total flow; pulse 5 kV, 20 kHz, 1 μs; sine 8.5 kV, 7.5 kHz; dome cell: 30 mg 5 wt.% Ni/MgAlO_x_ powder, 1.6 vol% CO_2_/6.4 vol% H_2_/Ar, 155 mL min⁻^1^ total flow, pulse 5 kV, 20 kHz, 1 μs). Data represent mean ± SD (n = 3).


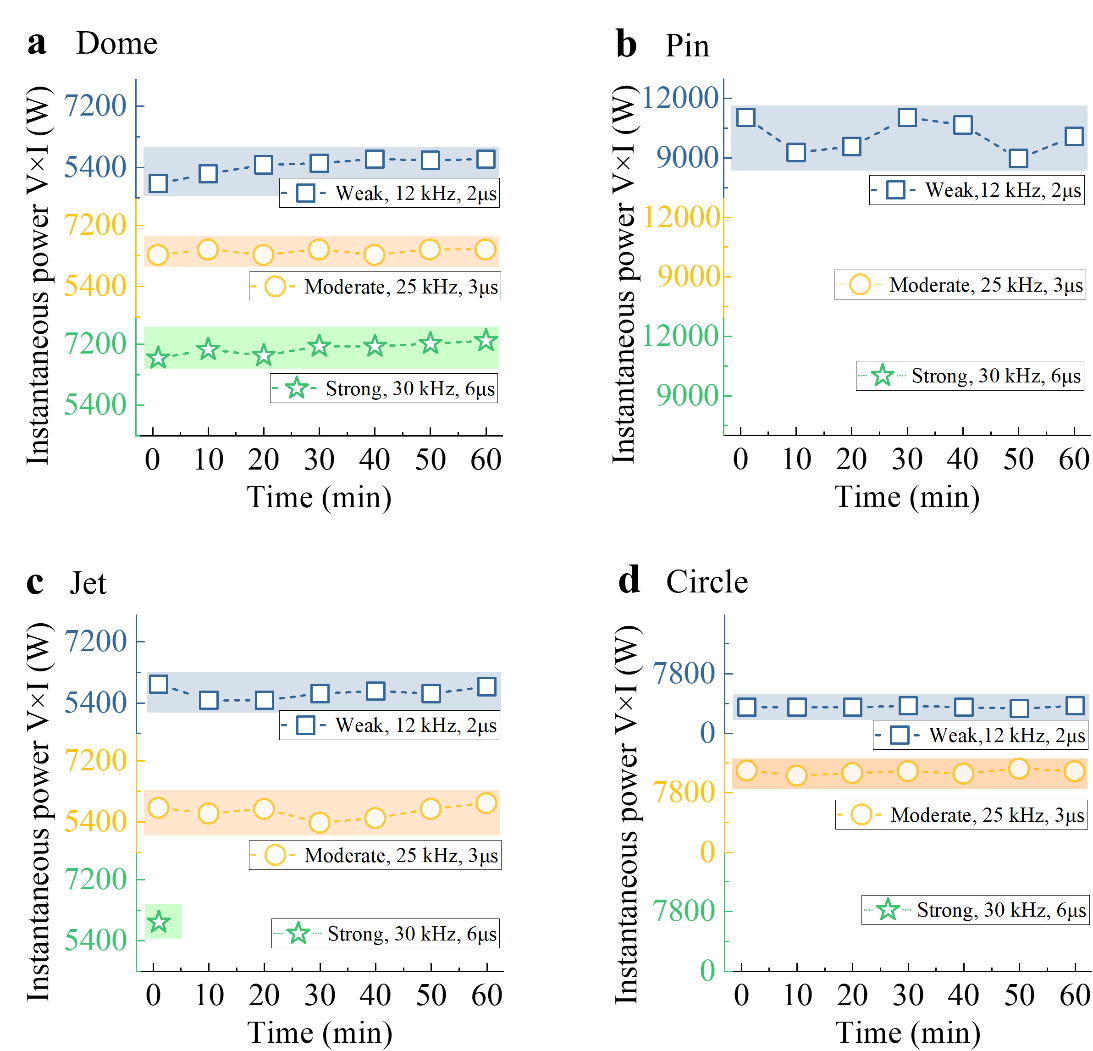


**Figure S16.** (a–d) Instantaneous power (V×I) of the dome, pin, jet, and circle cell under pulse excitation conditions of weak (12 kHz, 2 µs), moderate (25 kHz, 3 µs), and strong (30 kHz, 6 µs) input. All experiments were performed over the 5 wt.% Ni/MgAlO_x_ catalyst with a feed gas of 95 vol% Ar + 1 vol% CO_2_ + 4 vol% H_2_ at a total flow rate of 50 mL/min.


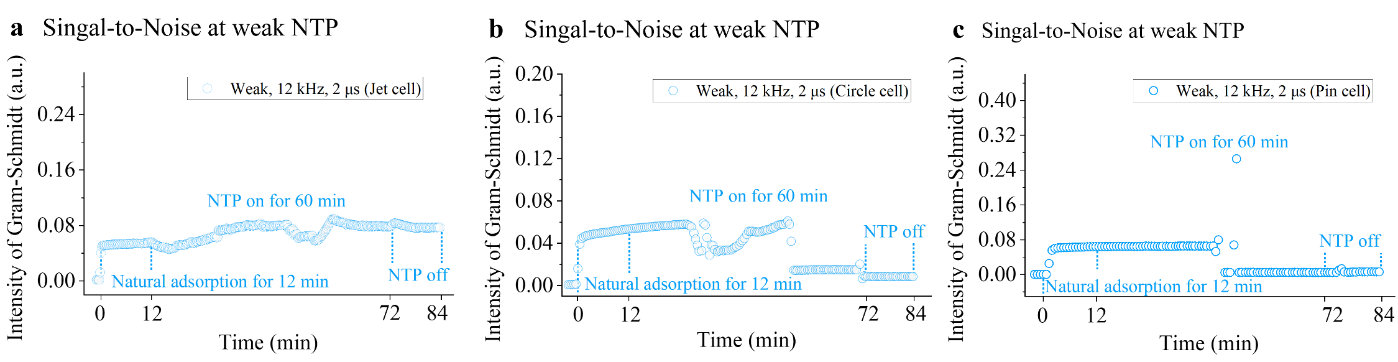


**Figure S17.** (a–c) Gram-Schmidt intensity of the jet-/circle-/pin-type cell under weak pulse excitation (12 kHz, 2 µs). All experiments were performed over the 5 wt.% Ni/MgAlO_x_ catalyst with a feed gas of 95 vol% Ar + 1 vol% CO_2_ + 4 vol% H_2_ at a total flow rate of 50 mL/min.


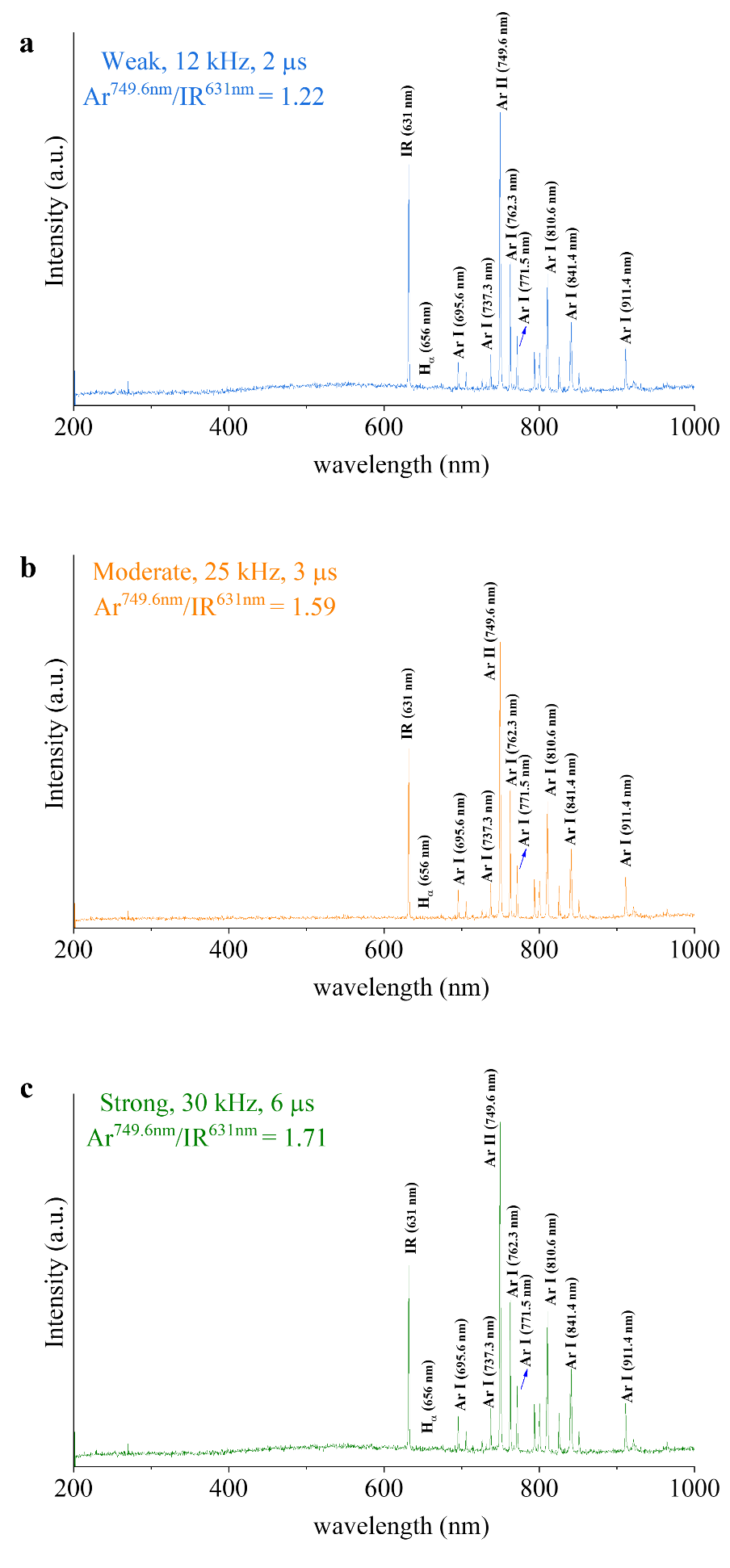


**Figure S18.** OES of the dome cell under different pulse plasma discharge intensities (a) 12 kHz, 2 µs; (b) 25 kHz, 3 µs; (c) 30 kHz, 6 µs.


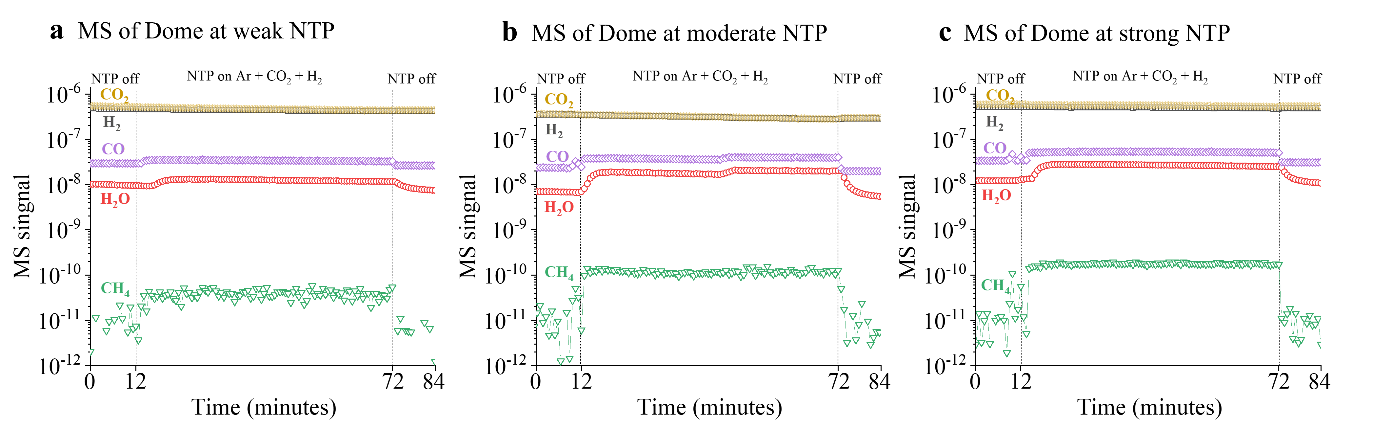


**Figure S19.** (a, b, c) MS signals were collected at the dome cell exit under pulse excitation conditions of weak (12 kHz, 2 μs), moderate (25 kHz, 3 μs), and strong (30 kHz, 6 μs) input. All experiments were performed over the 5 wt.% Ni/MgAlO_x_ catalyst with a feed gas of 95 vol% Ar + 1 vol% CO_2_ + 4 vol% H_2_ at a total flow rate of 50 mL/min.


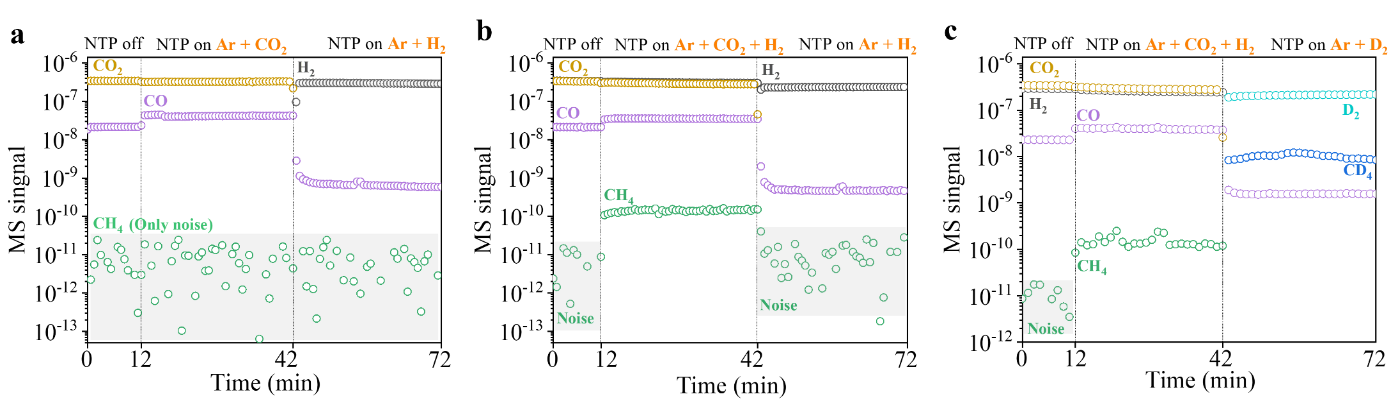


**Figure S20.** MS signals (m/z = 44 for CO_2_, 2 for H_2_, 4 for D_2_, 28 for CO, 18 for H_2_O, 15 for CH_4_, and 19 for CD_4_) collected at the flow cell exit in the gas-switching experiments of (a) from 99 vol% Ar + 1 vol% CO_2_ NTP to 96 vol% Ar + 4 vol% H_2_ NTP, (b) from 95 vol% Ar + 1 vol% CO_2_ + 4 vol% H_2_ NTP to 96 vol% Ar + 4 vol% H_2_ NTP, and (c) from 95 vol% Ar + 1 vol% CO_2_ + 4 vol% H_2_ NTP to 96 vol% Ar + 4 vol% D_2_ NTP, over the 5 wt.% Ni/MgAlO_x_ catalyst. Experiments in (a, b) were conducted under 5 kV, 20 kHz, 1 μs pulse input; experiment in (c) under 5 kV, 30 kHz, 6 μs.


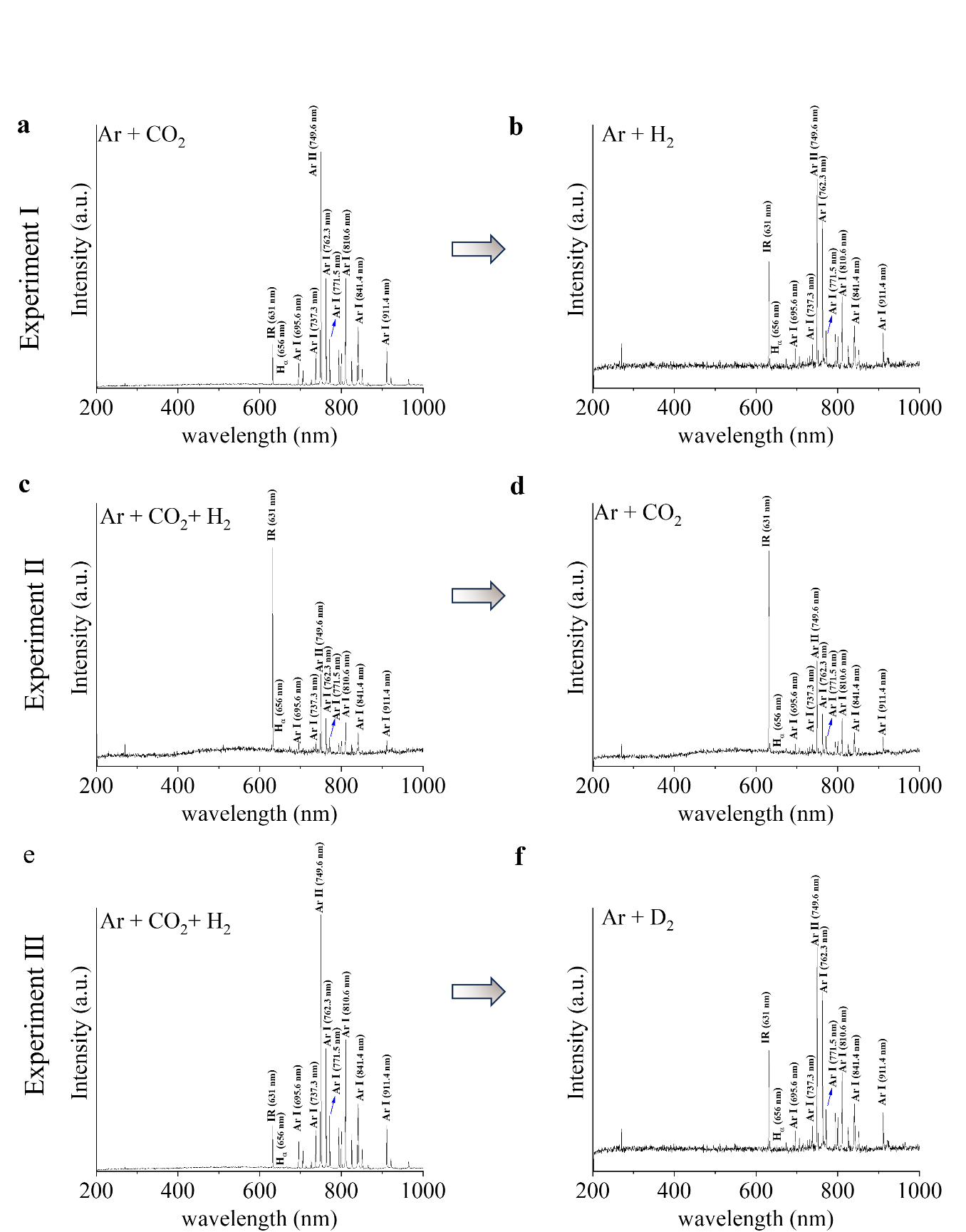


**Figure S21.** OES of the dome cell in the transition-state (a) from 99 vol% Ar + 1 vol% CO_2_ NTP to 96 vol% Ar + 4 vol% H_2_ NTP, (b) from 95 vol% Ar + 1 vol% CO_2_ + 4 vol% H_2_ NTP to 96 vol% Ar + 4 vol% H_2_ NTP, and (c) from 95 vol% Ar + 1 vol% CO_2_ + 4 vol% H_2_ NTP to 96 vol% Ar + 4 vol% D_2_ NTP, over the 5 wt.% Ni/MgAlO_x_ catalyst. Experiments in (a, b) were conducted under 5 kV, 20 kHz, 1 μs pulse input; experiment in (c) under 5 kV, 30 kHz, 6 μs; atmospheric pressure; bulk cell temperature ∼54–60 °C.
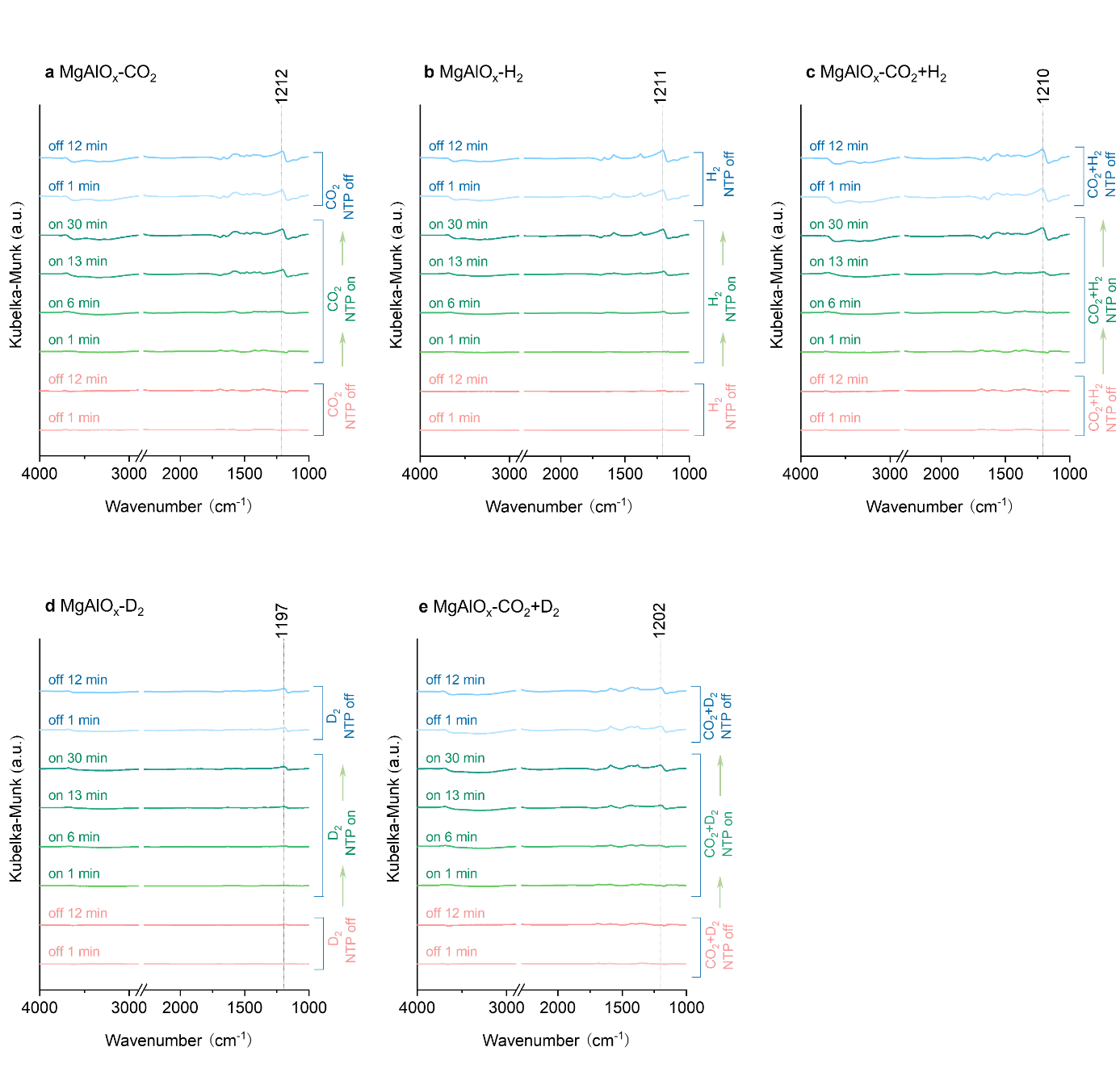


**Figure S22.** In-situ DRIFTS spectra of the bare MgAlO_x_ support under gas atmospheres of (a) CO_2_, (b) H_2_, (c) CO_2_ + H_2_, (d) D_2_, and (e) CO_2_ + D_2_ with and without plasma excitation (NTP on–off–on sequences). The weak band observed at 1197–1212 cm⁻^1^ corresponds to δ(OH) vibration, attributed to surface hydroxyl groups.


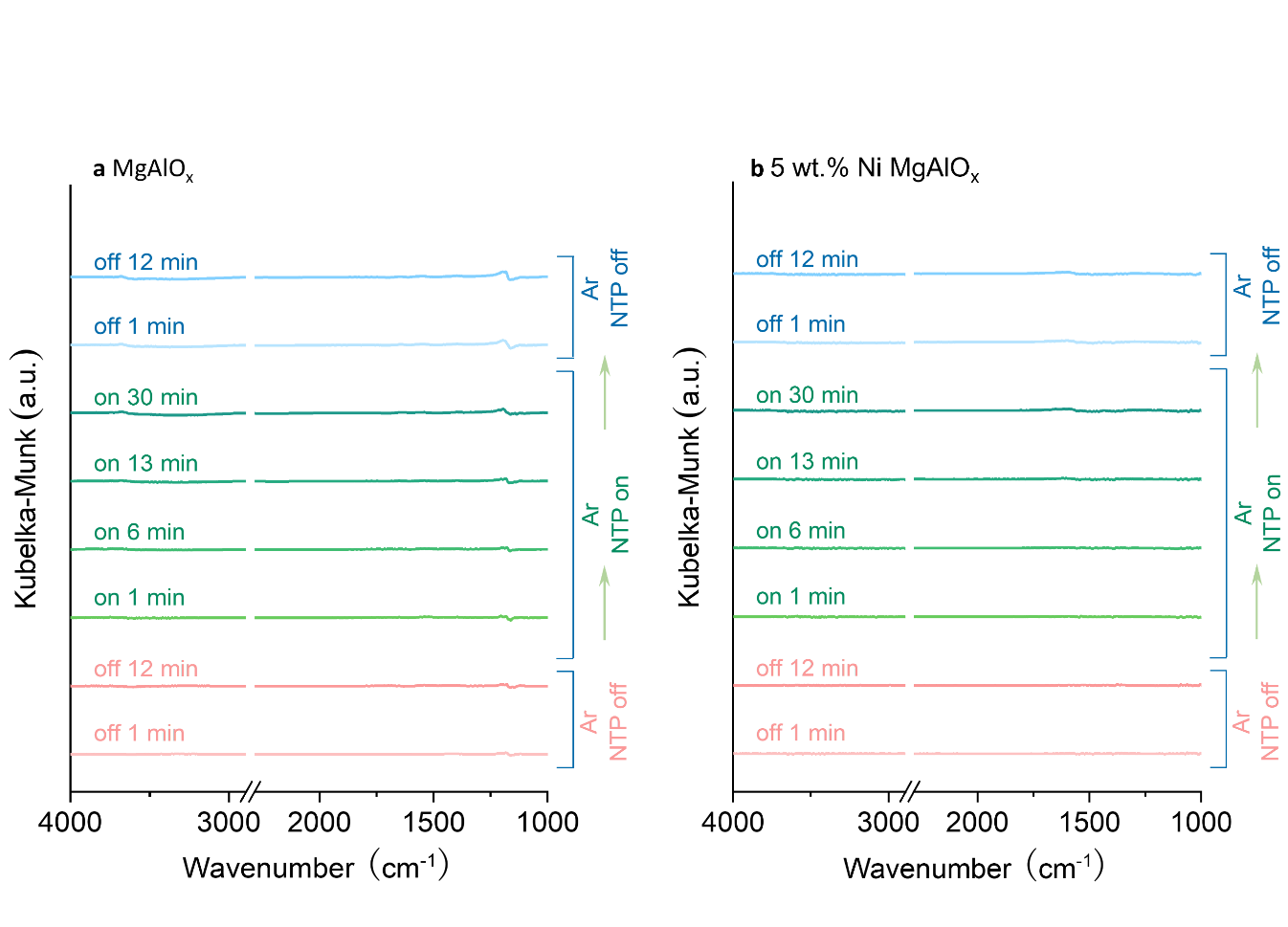


**Figure S23.** In-situ DRIFTS spectra of (a) MgAlO_x_ and (b) 5 wt.% Ni/MgAlO_x_ under pure Ar atmosphere with plasma off–on–off cycles.


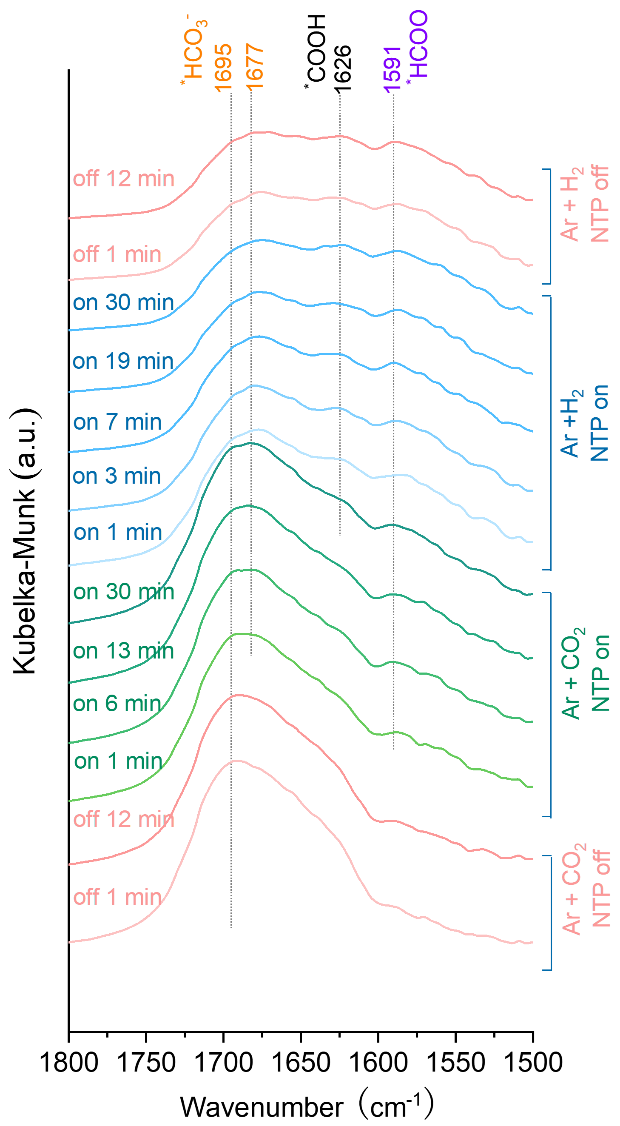


**Figure S24.** Zoomed in at 1500-1800 cm⁻^1^ of DRIFTS spectra of the dome cell in the transition-state from 99 vol% Ar + 1 vol% CO_2_ NTP (p_CO2_ = 0.01 atm) to 96 vol% Ar + 4 vol% H_2_ NTP (p_H2_ = 0.04 atm).


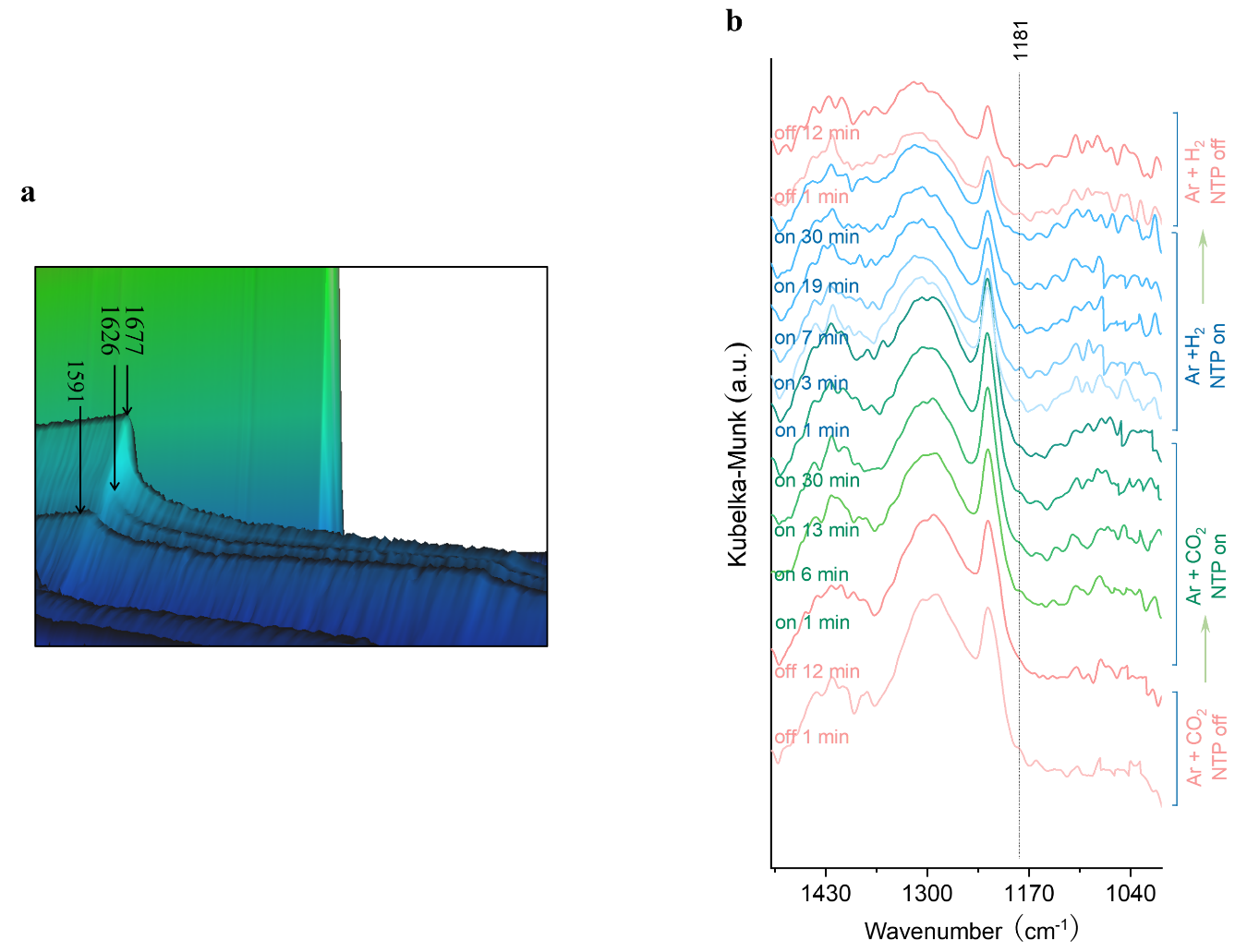


**Figure S25.** Evolution of the IR spectra upon plasma gas switching from 99 vol% Ar + 1 vol% CO_2_ to 96 vol% Ar + 4 vol% H_2_ (the experiment I): (a) The new bands at 1626 cm⁻^1^ and (b) 1181 cm⁻^1^ emerged rapidly after plasma ignition under 5 kV, 20 kHz, and 1 μs pulse input, indicating the formation of surface ^*^COOH intermediates associated with gas-phase hydrogen species (via the E–R-like pathways).


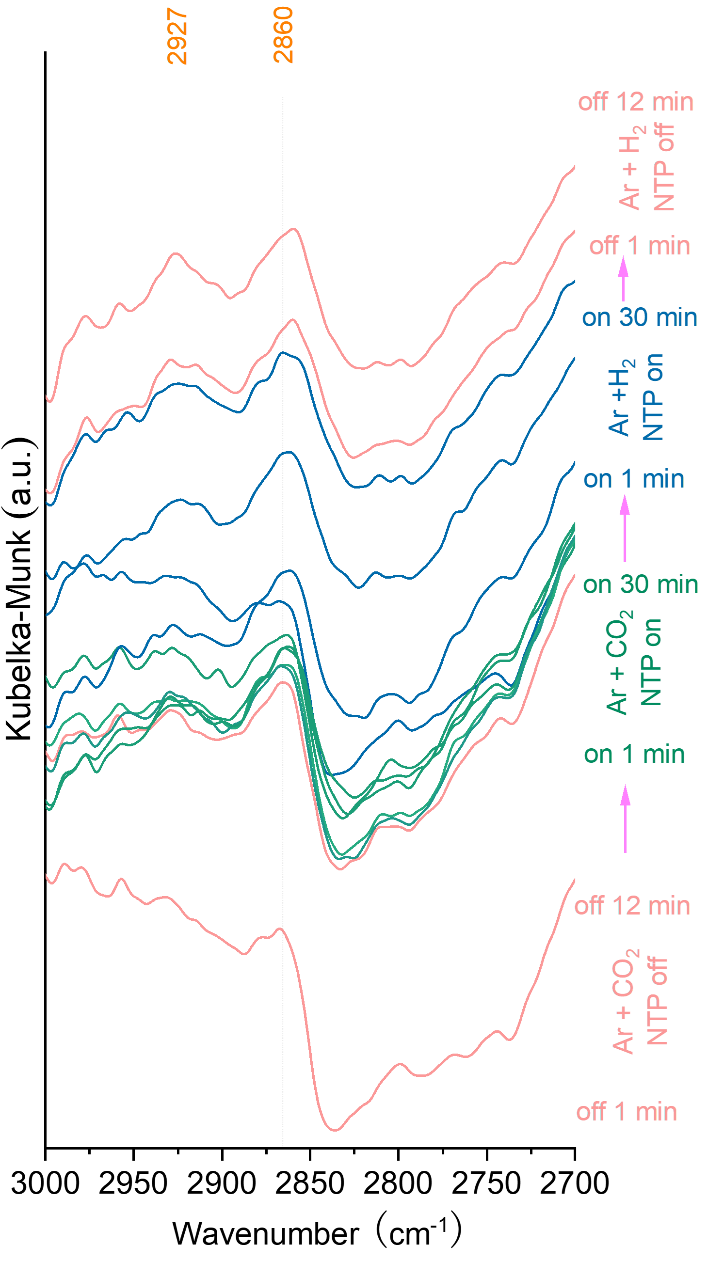


**Figure S26.** Zoomed in at 2700-3000 cm⁻^1^ of DRIFTS spectra of the dome cell in the transition-state from 99 vol% Ar + 1 vol% CO_2_ NTP (p_CO2_ = 0.01 atm) to 96 vol% Ar + 4 vol% H_2_ NTP (p_H2_ = 0.04 atm).


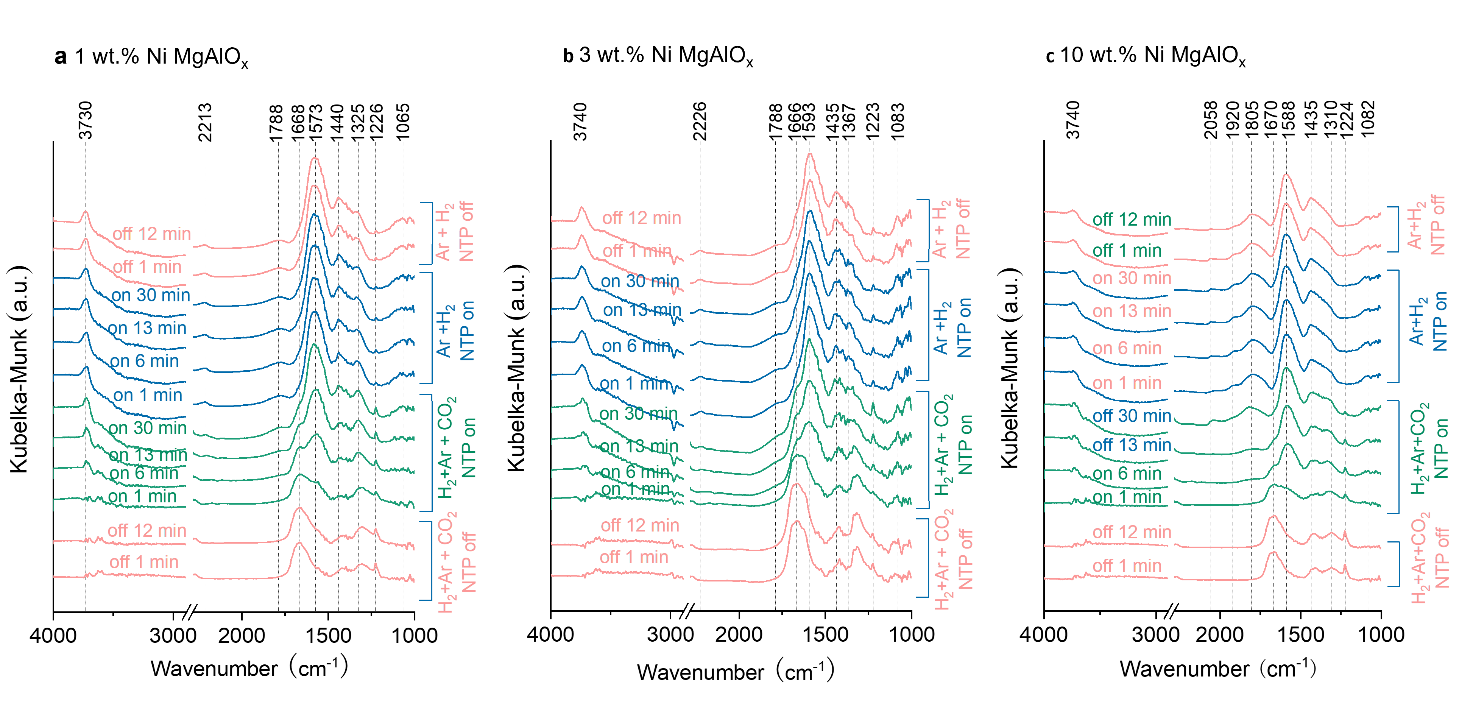


**Figure S27.** DRIFTS spectra of the dome cell in the transition-state from 95 vol% Ar + 1 vol% CO_2_ + 4 vol% H_2_ NTP (p_CO2_ = 0.01 atm, p_H2_ = 0.04 atm) to 96 vol% Ar + 4 vol% H_2_ NTP (p_H2_ = 0.04 atm) of (a) 1 wt.% Ni MgAlO_x_, (b) 3 wt.% Ni MgAlO_x_, (c) 10 wt.% Ni MgAlO_x_.


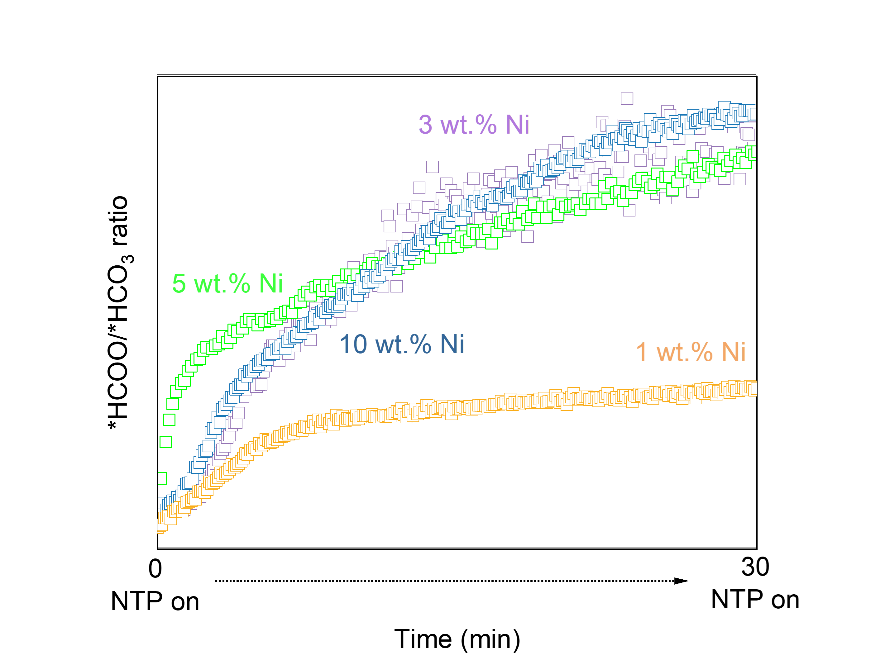


**Figure S28.** The trend of ^*^HCOO/^*^HCO_3_ of DRIFTS spectra from different Ni loading amounts in the dome cell in the transition-state from NTP off to NTP on at 95 vol% Ar + 1 vol% CO_2_ + 4 vol% H_2_ (p_CO2_ = 0.01 atm, p_H2_ = 0.04 atm).


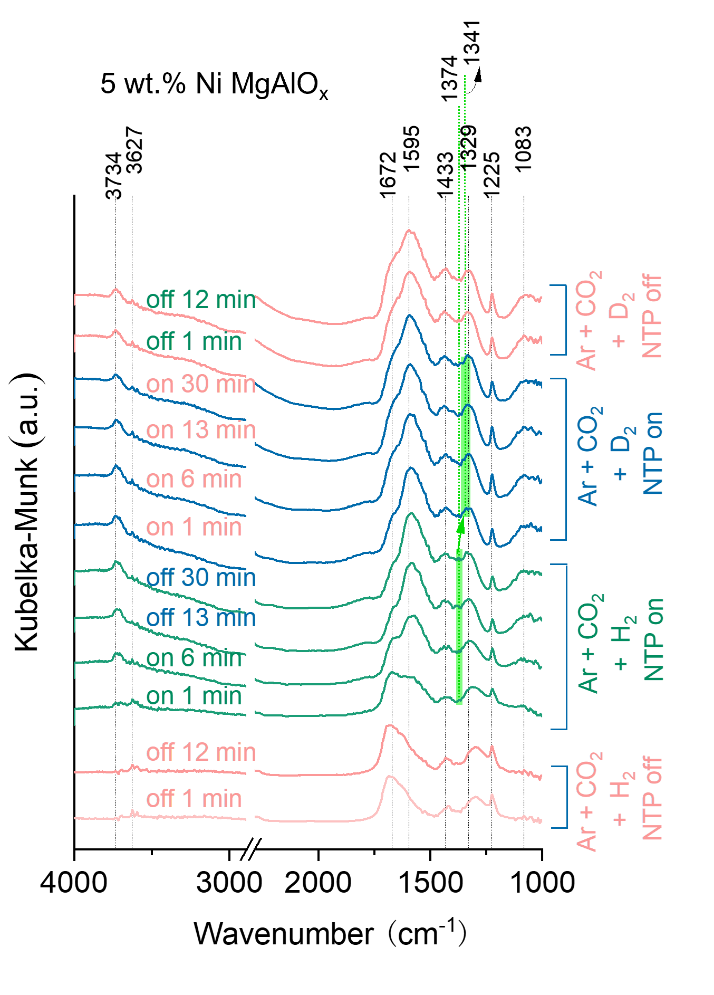


**Figure S29.** DRIFTS spectra of the dome cell in the transition-state (c) from 95 vol% Ar + 1 vol% CO_2_ + 4 vol% H_2_ NTP (p_CO2_ = 0.01 atm, p_H2_ = 0.04 atm) to 95 vol% Ar + 1 vol% CO_2_ + 4 vol% D_2_ NTP (p_CO2_ = 0.01 atm, p_D2_ = 0.04 atm), over the 5 wt.% Ni/MgAlO_x_ catalyst.


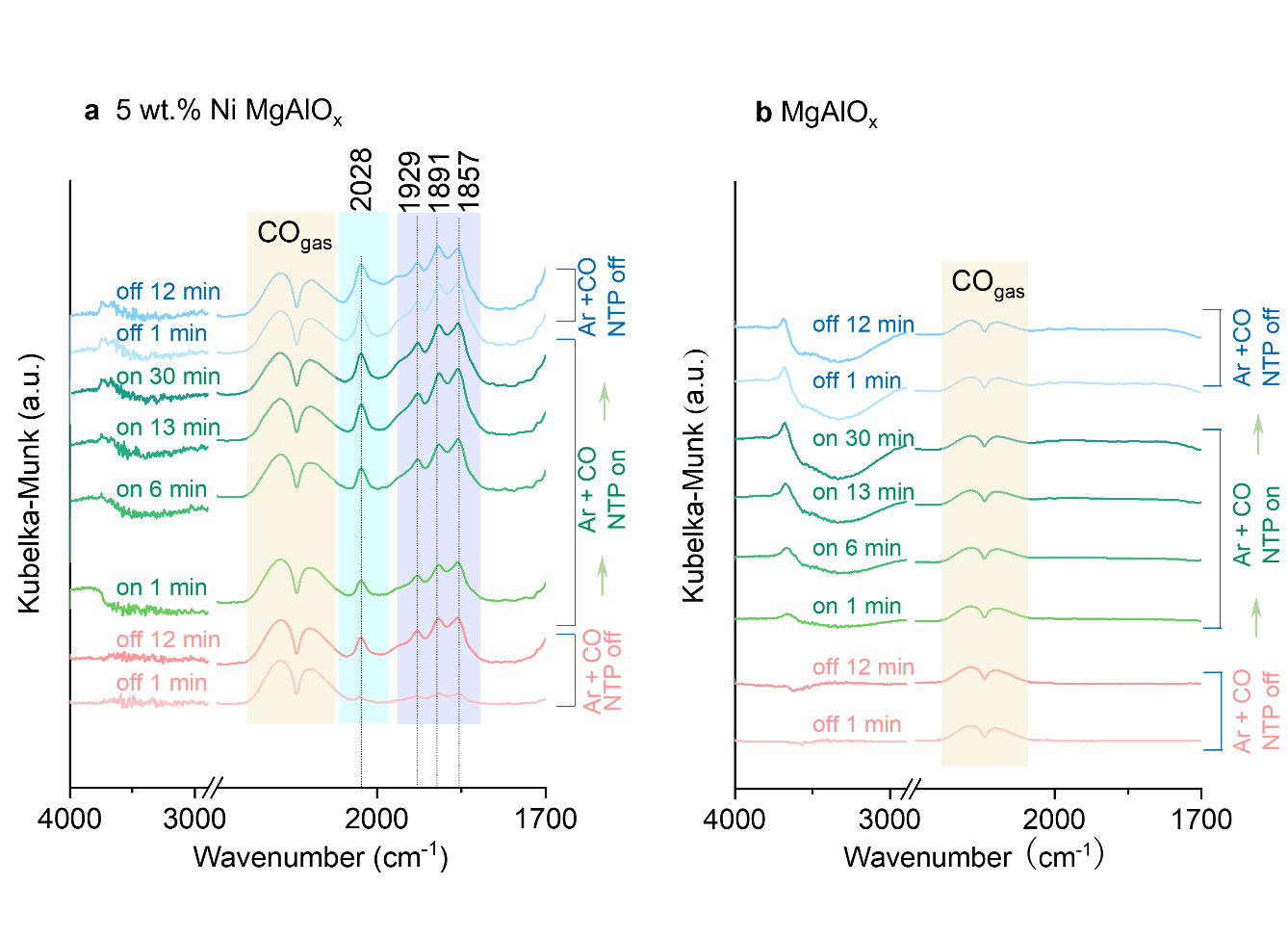


**Figure S30.** In-situ DRIFTS spectra of (a) 5 wt.% Ni/MgAlO_x_ and (b) bare MgAlO_x_ support under CO flow during the NTP on–off–on sequence. Distinct linearly adsorbed CO (2028 cm⁻^1^) and bridged CO (1857, 1891, 1929 cm⁻^1^) bands are observed for the 5 wt.% Ni/MgAlO_x_, while only gas-phase CO peaks are detected on the bare support.

**Supplementary References**

1 Hong, Y. *et al.* Electron temperature and density measurement of a dielectric barrier discharge argon plasma generated with tube-to-plate electrodes in water. *Vacuum* **130**, 130-136 (2016). https://doi.org/10.1016/j.vacuum.2016.05.012

2 Subedi, D. P. *et al.* Investigation on Parameters of Atmospheric Pressure Plasma Jet by Electrical and Optical Methods. *Journal of Nepal Physical Society* **6**, 50-56 (2020). https://doi.org/10.3126/jnphyssoc.v6i2.34857

3 Amamou, H., Escarguel, A. & Ferhat, B. Diagnostics of inhomogeneous plasmas: correction coefficients of the self-absorption and of the effect of spatial inhomogeneity. *Journal of Plasma Physics* **82** (2016). https://doi.org/10.1017/s0022377816000283

4 Chen, S. *et al.* Optimization of nonthermal plasma (NTP) catalytic CO2 methanation: effect of the excitation waveform, pellet size and residence time. *Reaction Chemistry & Engineering* (2025). https://doi.org/10.1039/d4re00628c

5 Zhou, Z. *et al.* Indium-Promoted ZnZrOx Solid Solution Catalyst for CO2 Hydrogenation to Methanol. *Industrial & Engineering Chemistry Research* **63**, 9026-9037 (2024). https://doi.org/10.1021/acs.iecr.4c00953

6 Wu, X. *et al.* Insight into the impacts of MnO2 crystal phases on CO2 selective hydrogenation to CH4 on Ni/MnOx catalysts. *Journal of CO2 Utilization* **75** (2023). https://doi.org/10.1016/j.jcou.2023.102584

7 Etim, U. J., Zhang, C. & Zhong, Z. Impacts of the Catalyst Structures on CO(2) Activation on Catalyst Surfaces. *Nanomaterials (Basel)* **11** (2021). https://doi.org/10.3390/nano11123265

8 Suksumrit, K., Kleiber, S. & Lux, S. The Role of Carbonate Formation during CO2 Hydrogenation over MgO-Supported Catalysts: A Review on Methane and Methanol Synthesis. *Energies* **16** (2023). https://doi.org/10.3390/en16072973

9 Xu, S. *et al.* CO Poisoning of Ru Catalysts in CO2 Hydrogenation under Thermal and Plasma Conditions: A Combined Kinetic and Diffuse Reflectance Infrared Fourier Transform Spectroscopy–Mass Spectrometry Study. *ACS Catalysis* **10**, 12828-12840 (2020). https://doi.org/10.1021/acscatal.0c03620

10 Kim, H.-H., Ogata, A. & Futamura, S. Oxygen partial pressure-dependent behavior of various catalysts for the total oxidation of VOCs using cycled system of adsorption and oxygen plasma. *Applied Catalysis B: Environmental* **79**, 356-367 (2008). https://doi.org/10.1016/j.apcatb.2007.10.038

11 Kim, D. Y. *et al.* Cooperative Catalysis of Vibrationally Excited CO2 and Alloy Catalyst Breaks the Thermodynamic Equilibrium Limitation. *J Am Chem Soc* **144**, 14140-14149 (2022). https://doi.org/10.1021/jacs.2c03764

12 Cui, Z. *et al.* Plasma-Catalytic Methanol Synthesis from CO2 Hydrogenation over a Supported Cu Cluster Catalyst: Insights into the Reaction Mechanism. *ACS Catalysis* **12**, 1326-1337 (2022). https://doi.org/10.1021/acscatal.1c04678

13 Li, S. *et al.* Tuning the CO(2) Hydrogenation Selectivity of Rhodium Single-Atom Catalysts on Zirconium Dioxide with Alkali Ions. *Angew Chem Int Ed Engl* **62**, e202218167 (2023). https://doi.org/10.1002/anie.202218167

14 Xu, S. *et al.* Sustaining metal–organic frameworks for water–gas shift catalysis by non-thermal plasma. *Nature Catalysis* **2**, 142-148 (2019). https://doi.org/10.1038/s41929-018-0206-2

15 Chen, H. *et al.* Coupling non-thermal plasma with Ni catalysts supported on BETA zeolite for catalytic CO2 methanation. *Catalysis Science & Technology* **9**, 4135-4145 (2019). https://doi.org/10.1039/c9cy00590k

16 Chen, H. *et al.* Nonthermal plasma (NTP) activated metal–organic frameworks (MOFs) catalyst for catalytic CO2 hydrogenation. *AIChE Journal* **66** (2019). https://doi.org/10.1002/aic.16853

17 Xu, S. *et al.* Mechanistic study of non-thermal plasma assisted CO2 hydrogenation over Ru supported on MgAl layered double hydroxide. *Applied Catalysis B: Environmental* **268** (2020). https://doi.org/10.1016/j.apcatb.2020.118752

18 Stere, C. *et al.* A design of a fixed bed plasma DRIFTS cell for studying the NTP-assisted heterogeneously catalysed reactions. *Catalysis Science & Technology* **10**, 1458-1466 (2020). https://doi.org/10.1039/d0cy00036a

19 Wu, K. *et al.* Tuning the local electronic structure of SrTiO(3) catalysts to boost plasma-catalytic interfacial synergy. *J Hazard Mater* **428**, 128172 (2022). https://doi.org/10.1016/j.jhazmat.2021.128172

20 Turan, N., Barboun, P. M., Nayak, P. K., Hicks, J. C. & Go, D. B. Development of a small-scale helical surface dielectric barrier discharge for characterizing plasma–surface interfaces. *Journal of Physics D: Applied Physics* **53** (2020). https://doi.org/10.1088/1361-6463/ab8320

21 Meng, S. *et al.* Plasma‐driven CO2 hydrogenation to CH3OH over Fe2O3/γ‐Al2O3 catalyst. *AIChE Journal* **69** (2023). https://doi.org/10.1002/aic.18154

22 Stere, C. E. *et al.* Probing a Non-Thermal Plasma Activated Heterogeneously Catalyzed Reaction Using in Situ DRIFTS-MS. *ACS Catalysis* **5**, 956-964 (2015). https://doi.org/10.1021/cs5019265

23 Zhang, S., Li, Y., Knoll, A. & Oehrlein, G. S. Mechanistic aspects of plasma-enhanced catalytic methane decomposition by time-resolved operando diffuse reflectance infrared Fourier transform spectroscopy. *Journal of Physics D: Applied Physics* **53** (2020). https://doi.org/10.1088/1361-6463/ab795b

24 Zhang, X. *et al.* Boosting methanol productionviaplasma catalytic CO2hydrogenation over a MnOx/ZrO2catalyst. *Catalysis Science & Technology* **13**, 2529-2539 (2023). https://doi.org/10.1039/d2cy02015g

25 Rodrigues, A., Tatibouët, J.-M. & Fourré, E. Operando DRIFT Spectroscopy Characterization of Intermediate Species on Catalysts Surface in VOC Removal from Air by Non-thermal Plasma Assisted Catalysis. *Plasma Chemistry and Plasma Processing* **36**, 901-915 (2016). https://doi.org/10.1007/s11090-016-9718-1

26 Sheng, Z., Kim, H.-H., Yao, S. & Nozaki, T. Plasma-chemical promotion of catalysis for CH4dry reforming: unveiling plasma-enabled reaction mechanisms. *Physical Chemistry Chemical Physics* **22**, 19349-19358 (2020). https://doi.org/10.1039/d0cp03127e

27 Parastaev, A., Kosinov, N. & Hensen, E. J. M. Mechanistic study of catalytic CO2 hydrogenation in a plasma by operando DRIFT spectroscopy. *Journal of Physics D: Applied Physics* **54** (2021). https://doi.org/10.1088/1361-6463/abeb96

28 Wang, Y. *et al.* Engineering Ni-Co bimetallic interfaces for ambient plasma-catalytic CO2 hydrogenation to methanol. *Chem* **10**, 2590-2606 (2024). https://doi.org/10.1016/j.chempr.2024.06.022

29 Xu, S., et al. Product selectivity controlled by the nano-environment of Ru ZSM-5 catalysts in nonthermal plasma catalytic CO2 hydrogenation. *Applied Catalysis B: Environment and Energy* **348** 123826 (2024). https://doi.org/10.1016/j.apcatb.2024.123826

30 Liu, X. *et al.* Atomically Thick Oxide Overcoating Stimulates Low-Temperature Reactive Metal-Support Interactions for Enhanced Catalysis. *J Am Chem Soc* **145**, 6702-6709 (2023). https://doi.org/10.1021/jacs.2c12046

31 Mao, D., Zhang, H., Zhang, J. & Wu, D. The influence of the compositions and structures of Cu-ZrO2 catalysts on the catalytic performance of CO2 hydrogenation to CH3OH. *Chemical Engineering Journal* **471** (2023). https://doi.org/10.1016/j.cej.2023.144605

32 Rawool, S. A. *et al.* Direct CO(2) capture and conversion to fuels on magnesium nanoparticles under ambient conditions simply using water. *Chem Sci* **12**, 5774-5786 (2021). https://doi.org/10.1039/d1sc01113h

33 Li, Y. *et al.* Experimental and theoretical insights into an enhanced CO2 methanation mechanism over a Ru-based catalyst. *Applied Catalysis B: Environmental* **319** (2022). https://doi.org/10.1016/j.apcatb.2022.121903

34 Yang, Y. *et al.* Gallium Cluster-Promoted In2O3 Catalyst for CO2 Hydrogenation to Methanol. *ACS Catalysis* **14**, 13958-13972 (2024). https://doi.org/10.1021/acscatal.4c03045

35 Zou, S. *et al.* Structure-performance correlation on bimetallic catalysts for selective CO2 hydrogenation. *Energy & Environmental Science* **16**, 5513-5524 (2023). https://doi.org/10.1039/d3ee01650a

36 Tian, H. *et al.* Tandem composite of M (Zn, Ga, In)-UIO-66/(HZSM-5)-palygorskite for hydrogenation of carbon dioxide to aromatics. *Chemical Engineering Journal* **466** (2023). https://doi.org/10.1016/j.cej.2023.143267

37 Liu, D. *et al.* Twin S-Scheme g-C3N4/CuFe2O4/ZnIn2S4 Heterojunction with a Self-Supporting Three-Phase System for Photocatalytic CO2 Reduction: Mechanism Insight and DFT Calculations. *ACS Catalysis* **14**, 5326-5343 (2024). https://doi.org/10.1021/acscatal.4c00409

38 Wang, L. *et al.* Silica accelerates the selective hydrogenation of CO(2) to methanol on cobalt catalysts. *Nat Commun* **11**, 1033 (2020). https://doi.org/10.1038/s41467-020-14817-9

39 Hu, J. *et al.* Sulfur vacancy-rich MoS2 as a catalyst for the hydrogenation of CO2 to methanol. *Nature Catalysis* **4**, 242-250 (2021). https://doi.org/10.1038/s41929-021-00584-3

40 Wu, L. *et al.* Constructing Bridge Hydroxyl Groups on the Ru/MO(x)/HZSM-5 (M = W, Mo) Catalysts to Promote the Hydrolysis Oxidation of Multicomponent VOCs. *Environ Sci Technol* **59**, 945-955 (2025). https://doi.org/10.1021/acs.est.4c09649
